# Supplementary material for: Effect of fascial closure using barbed sutures on incisional hernias in midline laparotomy for gynecological diseases: A multicenter randomized controlled trial (KGOG 4001)
Source: PLoS One. 2025 Nov 19;20(11):e0337036. doi: 10.1371/journal.pone.0337036 (PMC12629448; doi:10.1371/journal.pone.0337036)
Supplement: S1 Table — (DOCX) [file pone.0337036.s001.docx]

| S1 Table. BPI-K score at baseline between experimental and control group | | | |
| --- | --- | --- | --- |
|  | Experimental (barbed suture)  n=67 | Control (non-barbed suture)  n=71 | p value |
| Total | 14.2 ± 22.6 | 11.1 ± 19.0 | 0.390 |
| Pain worst | 1.78 ± 2.6 | 1.4 ± 2.3 | 0.376 |
| Pain least | 0.7 ± 1.5 | 0.7 ± 1.4 | 0.991 |
| Pain average | 1.3 ± 1.9 | 1.1 ± 1.8 | 0.585 |
| Pain now | 1.1 ±1.9 | 0.9 ± 1.6 | 0.479 |
| Activity | 1.4 ± 2.6 | 1.2 ± 2.2 | 0.565 |
| Mood | 1.6 ± 2.6 | 1.3 ± 2.3 | 0.518 |
| Ambulation | 1.4 ± 2.6 | 0.9 ± 2.1 | 0.291 |
| Work | 1.2 ± 2.4 | 0.9 ± 1.9 | 0.396 |
| Relation | 0.9 ± 1.9 | 0.8 ± 1.7 | 0.693 |
| Sleep | 1.4 ± 2.5 | 1.0 ± 1.9 | 0.333 |
| Enjoy | 1.5 ± 2.4 | 0.9 ± 1.9 | 0.136 |
| Values are presented as mean ± standard deviation | | | |
