# Supplementary material for: Effect of fascial closure using barbed sutures on incisional hernias in midline laparotomy for gynecological diseases: A multicenter randomized controlled trial (KGOG 4001)
Source: PLoS One. 2025 Nov 19;20(11):e0337036. doi: 10.1371/journal.pone.0337036 (PMC12629448; doi:10.1371/journal.pone.0337036)
Supplement: S2 Table — (DOCX) [file pone.0337036.s002.docx]

| S2 Table. BPI-K score at postoperative day 2 between experimental and control group | | | |
| --- | --- | --- | --- |
|  | Experimental (barbed suture)  n=67 | Control (non-barbed suture)  n=71 | p value |
| Total | 54.0 ± 27.4 | 58.1 ± 24.9 | 0.361 |
| Pain worst | 6.4 ± 2.3 | 6.7 ± 2.2 | 0.408 |
| Pain least | 3.9 ± 2.4 | 4.1 ± 2.2 | 0.677 |
| Pain average | 5.0 ± 2.1 | 5.2 ± 2.0 | 0.566 |
| Pain now | 4.7 ± 2.4 | 5.0 ± 2.4 | 0.542 |
| Activity | 5.7 ± 2.7 | 5.7 ± 2.5 | 0.849 |
| Mood | 5.3 ± 2.8 | 5.6 ± 2.8 | 0.496 |
| Ambulation | 5.5 ± 2.7 | 5.7 ± 2.8 | 0.746 |
| Work | 5.7 ± 2.9 | 5.7 ± 2.8 | 0.925 |
| Relation | 4.6 ± 2.9 | 4.8 ± 2.7 | 0.758 |
| Sleep | 4.7 ± 2.9 | 5.0 ± 2.9 | 0.640 |
| Enjoy | 5.0 ± 3.0 | 5.6 ± 3.0 | 0.311 |
| Values are presented as mean ± standard deviation | | | |
