# Supplementary material for: Effect of fascial closure using barbed sutures on incisional hernias in midline laparotomy for gynecological diseases: A multicenter randomized controlled trial (KGOG 4001)
Source: PLoS One. 2025 Nov 19;20(11):e0337036. doi: 10.1371/journal.pone.0337036 (PMC12629448; doi:10.1371/journal.pone.0337036)
Supplement: S3 Table — (DOCX) [file pone.0337036.s003.docx]

| S3 Table. BPI-K score at postoperative day 4 between experimental and control group | | | |
| --- | --- | --- | --- |
|  | Experimental (barbed suture)  n=67 | Control (non-barbed suture)  n=71 | p value |
| Total | 36.4 ± 23.3 | 39.6 ± 24.0 | 0.431 |
| Pain worst | 4.5 ± 2.1 | 5.1 ± 2.6 | 0.187 |
| Pain least | 2.8 ± 2.1 | 3.0 ± 2.1 | 0.572 |
| Pain average | 3.4 ± 1.9 | 3.8 ± 1.9 | 0.290 |
| Pain now | 3.2 ± 1.9 | 3.5 ± 2.1 | 0.552 |
| Activity | 4.0 ± 2.4 | 4.3 ± 2.4 | 0.497 |
| Mood | 3.6 ± 2.5 | 4.1 ± 2.4 | 0.297 |
| Ambulation | 3.6 ± 2.4 | 4.1 ± 2.4 | 0.265 |
| Work | 3.8 ± 2.5 | 4.0 ± 2.4 | 0.738 |
| Relation | 3.1 ± 2.5 | 3.4 ± 2.2 | 0.493 |
| Sleep | 3.3 ± 2.7 | 3.6 ± 2.4 | 0.458 |
| Enjoy | 3.5 ± 2.6 | 3.9 ± 2.4 | 0.332 |
| Values are presented as mean ± standard deviation | | | |
