# Supplementary material for: Effect of fascial closure using barbed sutures on incisional hernias in midline laparotomy for gynecological diseases: A multicenter randomized controlled trial (KGOG 4001)
Source: PLoS One. 2025 Nov 19;20(11):e0337036. doi: 10.1371/journal.pone.0337036 (PMC12629448; doi:10.1371/journal.pone.0337036)
Supplement: S4 Table — (DOCX) [file pone.0337036.s004.docx]

| S4 Table. NRS pain score from surgery to postoperative day 4 between experimental and control group | | | |
| --- | --- | --- | --- |
|  | Experimental (barbed suture)  n=67 | Control (non-barbed suture)  n=71 | p value |
| OP day 7A-3P | 6.3 ± 1.5 | 5.4 ± 2.8 | 0.299 |
| OP day 3P-11P | 5.8 ± 2.3 | 6.4 ± 1.8 | 0.091 |
| OP day 11P-7A | 5.3 ± 2.4 | 5.5 ± 2.4 | 0.642 |
| POD #1 7A-3P | 5.3 ± 2.2 | 5.1 ± 2.4 | 0.487 |
| POD #1 3P-11P | 5.1 ± 2.4 | 5.1 ± 2.3 | 0.811 |
| POD #1 11P-7A | 4.9 ± 2.1 | 4.6 ± 2.1 | 0.309 |
| POD #2 7A-3P | 4.8 ± 2.0 | 4.6 ± 2.2 | 0.508 |
| POD #2 3P-11P | 4.6 ± 2.0 | 4.6 ± 2.0 | 0.937 |
| POD #2 11P-7A | 4.2 ± 1.9 | 4.4 ± 1.9 | 0.543 |
| POD #3 7A-3P | 3.9 ± 1.9 | 4.1 ± 2.0 | 0.625 |
| POD #3 3P-11P | 3.7 ± 1.9 | 4.3 ± 2.0 | 0.111 |
| POD #3 11P-7A | 3.8 ± 1.8 | 3.7 ± 1.7 | 0.711 |
| POD #4 7A-3P | 3.3 ± 1.6 | 3.6 ± 1.7 | 0.298 |
| POD #4 3P-11P | 3.6 ± 1.9 | 3.7 ± 1.9 | 0.656 |
| Values are presented as mean ± standard deviation  NRS, Numeric Rating Scale; OP, operative; POD, postoperative day | | | |
