# Supplementary material for: Effect of fascial closure using barbed sutures on incisional hernias in midline laparotomy for gynecological diseases: A multicenter randomized controlled trial (KGOG 4001)
Source: PLoS One. 2025 Nov 19;20(11):e0337036. doi: 10.1371/journal.pone.0337036 (PMC12629448; doi:10.1371/journal.pone.0337036)

**Effect of Barbed Suture Fascia Closure on Incisional hernia in Midline Laparotomy for Gynecological Diseases (BARBHER)**

**Version No: 0.4**

**Principal Investigator’s Institution: Seoul National University Bundang Hospital**

**Principal Investigator’s Name: Kim, Ki-dong**

**Overview**

| Protocol Title | (Korean) 부인과질환으로 정중절개 개복술을 받는 여성에서 미늘봉합사를 이용한 근막봉합이 절개창탈장 발생에 미치는 영향 |
| --- | --- |
|  | (English) Effect of Barbed Suture Fascia Closure on Incisional hernia in Midline Laparotomy for Gynecological Diseases (BARBHER) |
| Principal Investigator | Professor Kim, Ki Dong, Department of Gynecology |
| Sponsor | Johnson & Johnson |

| Study Objective | To find out whether barbed suture fascia closure reduces incisional hernia in midline laparotomy for gynecological diseases |
| --- | --- |
| Study Design | Prospective, multi-center, randomized clinical study |
| Study Period | 40 months from the IRB approval date |
| Subject | Female patients who underwent midline laparotomy for gynecological diseases |
| Number of Subjects | 174 |
| Vulnerable Subjects | Vulnerable subjects do not participate in this study |
| Investigational Product/Medical Device | STRATAFIX Symmetric PDS Plus (SS-PDS), suture size: 1/0, suture length: 45cm, needle size: 40mm (CT needle) (Ethicon, Somerville, NJ, USA) |
| Dosage Regime | Fascia closure according to the instructions provided by the manufacturer |
| Study Method | Patients will be randomized to SS-PDS experimental group and control group at a 1:1 ratio:  SS-PDS experimental group: fascia closure using barbed suture (SS-PDS)  SS-PDS control group: fascia closure using conventional suture instead of barbed suture  Comparison of experimental vs. control group for 1-year cumulative incidence of incisional hernia.  In addition, surgical site infection, pain, adverse events will be compared.  Also as a secondary objective, patients will be randomized according to whether or not subcutaneous drain is performed and the results will be compared. |
| Major Inclusion Criteria | 1. Female patients planning to undergo elective midline laparotomy for gynecological diseases.  2. Over 18 years old.  3. ECOG performance status 0 - 2 |
| Major Exclusion Criteria | 1. Patients with previous or present abdominal incisional hernia.  2. Pregnant patients.  3. Patients receiving external radiation treatment to the pelvis or abdomen.  4. Patients allergic to PDS components.  5. Patient with diseases that affect wound healing including uncontrolled diabetes, autoimmune vasculitis, and hepatocirrhosis.  6. BMI>35  7. Patients are excluded if they have used or are expected to use drugs that may affect wound healing such as bevacizumab. In case where bevacizumab is used, participation is possible if they have a four-week washout period before and after surgery, respectively.  8. Patients who have had an abdominal midline laparotomy for the last 6 months.  9. If patients had surgery for suspected infection. |
| Efficacy Assessment | The efficacy of SS-PDS will be confirmed if 1-year cumulative incidence of incisional hernia in the experimental group is lower than that of the SS-PDS control group (p value 0.05) |
| Safety Assessment | Comparison of prevalence and grade of adverse events between SS-PDS experimental group and control group. |
| Schedule of Visit/Inspection | Baseline: eligibility check  Surgery: fascia closure with SS-PDS or conventional suture, subcutaneous drain insertion, collecting surgery-relevant parameters, pain level survey after surgery  4 weeks after surgery: surgical site infections and wound dehiscence are studied.  1 year after surgery: incisional hernia is judged. |
| Statistical Methods | Comparison of incidence of incisional hernia between SS-PDS experimental group and control group using Chi-square or Fisher’s exact test. |
| Expected Effects and Results | It is expected that the standard surgical method will be changed to fascia closure using barbed suture.  Incidence of incisional hernia is reduced to improve patients’ quality of life and reduce medical costs. |

**Protocol Synopsis**

1. **Protocol Title**

Effect of Barbed Suture Fascia Closure on Incisional hernia in Midline Laparotomy for Gynecological Diseases (BARBHER)

1. **Names and Addresses of Participating Sites and Principal Investigators by Institution**

| Participating Site (random order) | Address | Principal Investigator |
| --- | --- | --- |
| Seoul Nat’l Univ. Bundang Hospital | 82, Gumi-ro 173beon-gil, Bundang-gu, Seongnam-si, Gyeonggi-do | Kim, Ki-dong (Gynecology) |
| Samsung Medical Center | 81, Irwon-ro, Gangnam-gu, Seoul | Choi, Cheol-hoon (Gynecology) |
| National Cancer Center | 323, Ilsan-ro, Ilsandong-gu, Goyang-si, Gyeonggi-do | Yim, Myeong-cheol (Uterine & Ovarian Cancer Center) |
| Severance Hospital | 50-1, Yonsei-ro, Seodaemun-gu, Seoul | Lee, Jeong-yoon (Gynecology) |
| Asan Medical Center | 88, Olympic-ro 43-gil, Songpa-gu, Seoul | Park, Jeong-yeol (Gynecology) |
| Gachon Univ. Gil Medical Center | 21, Namdong-daero 774beon-gil, Namdong-gu, Incheon | Lee, Gwang-beom (Gynecology) |
| Ajou Univ. Hospital | 164, World cup-ro, Yeongtong-gu, Suwon-si, Gyeonggi-do | Jang, Seok-joon (Gynecology) |

* Prof. Kim, Ki-dong of Seoul Nat’l Univ. Bundang Hospital is a coordinating principal investigator.

1. **Names and Titles of Principal Investigator and Co-investigators**
2. **Principal Investigator**

Kim, Ki-dong (Associate Professor, Gynecology)

1. **Co-investigators**

Kim, Yong-beom (Professor, Gynecology)

Noh, Jae-hong (Associate Professor, Gynecology)

Seo, Dong-hoon (Associate Professor, Gynecology)

Kim, Joo-hyeon (Clinical Professor, Gynecology)

Kim, Joo-young (Full-time Doctor, Gynecology)

Hwang, Woo-yeon (Full-time Doctor, Gynecology)

1. **Research Associate**

Oh, Yoo-min (Research Nurse, Gynecology)

1. **Clinical Trial Pharmacist for Investigational Product / Manager of Investigational Medical Device**

Medical Device R&D Center, Seoul Nat’l Univ. Bundang Hospital

1. **Sponsor
   1) Name and Address of Sponsor:** None
   **2) Name(s) and Title(s) of Monitors:** CRAs of Korean Gynecologic Oncology Group (KGOG)
2. **Name and Address of Research Funding Institution**
3. **Name:** Johnson and Johnson
4. **Address:** New Brunswick, New Jersey
5. **RFP ID Number**: 2019-SFX-GEN-02
6. **Estimated Trial Period**

40 months from the date of IRB approval.

1. **Trial Disease**

Gynecological diseases that require midline laparotomy

1. **Background and Objectives**
2. **Background**

Incisional hernia is a type of hernia that forms at the site of surgical incision [PMID: 19495920]. Incisional hernia is a common complication of abdominal surgery with an incidence of 10-23%. The incidence is known to reach as high as 38% in high-risk group patients of old age, obesity, chronic obstructive pulmonary diseases, malnutrition, and abdominal aneurysm, etc. [PMID: 21254041]. Operative risk factors influential to the incidence of incisional hernia are incision types, sizes and abdominal closure [PMID:10195729, 3159324, 3954314, 2804595]. Incisional hernia degrades the quality of life and incurs a lot of cost [PMID:26206646].

Appropriate methods of abdominal fascia closure are suggested in various reviews and guidelines [PMID10714638, 15796944, 12594682, 11379640, 9926810, 20395846]. According to meta-analysis, incisional hernia incidence was lower with running suture using monofilament suture than interrupted suture because it is absorbed slower [PMID: 20395846,12594682, 26188742, 19917943]. However, more than 10% of the patients still experience incisional hernia after abdominal surgery even when adequate sutures and methods are used [PMID: 26188742]. Therefore, it is necessary to improve the method of abdominal fascia closure.

Barbed suture is a type of suture that has barbs on its surface. In many cases, similar or better closure results are obtained with barbed suture than those without barbs [PMID: 28603661]. STRATAFIX Symmetric PDS Plus (SS-PDS) is one of the barbed sutures that can be used for closure of high pressure sites such as fascia. SS-PDS demonstrates superior or similar results compared to conventional sutures without barbs in terms of strength and the ability to hold the tissues tight [PMID: 28603661].

Opinions vary as to whether subcutaneous drain insertion at the time of abdominal closure reduces wound dehiscence [PMID: 19442311]. Several studies report that subcutaneous drain insertion reduces wound complications [PMID: 24952366].

**2) Trial Hypothesis and Purpose**

(1) Schema


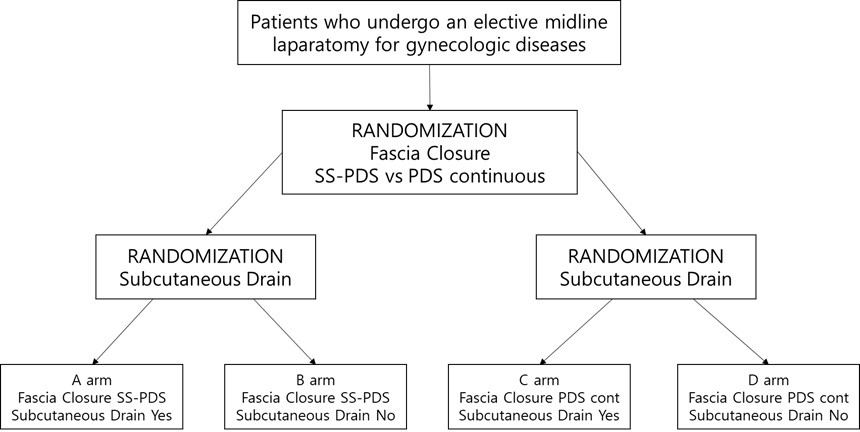


(2) Primary Objective

The purpose of this trial is to verify the hypothesis: ‘In comparison to sutures without barbs, using SS-PDS for abdominal fascia closure on female patients who undergo incisional hernia in midline laparotomy due to gynecological diseases will reduce the cumulative incidence of incisional hernia for 1 year after surgery.’

(3) Secondary Objective

A. Compare the incidence curves of incisional hernia over time between A+B arms and C+D arms (depending on whether SS-PDS is used or not).

B. Compare cumulative incidence and types of surgical site infections up to 4 weeks after surgery between A+B arms and C+D arms (depending on whether SS-PDS is used or not).

C. Compare incidence and cumulative incidence of wound dehiscence at Week 4 after surgery between A+B arms and C+D arms.

D. Compare BPI-K scores that are obtained on Day 2 after surgery between A+B arms and C+D arms (depending on whether SS-PDS is used or not)

E. Compare BPI-K scores that are obtained on Day 4 after surgery between A+B arms and C+D arms (depending on whether SS-PDS is used or not)

F. Compare NRS scores that are collected until Day 4 after surgery between A+B arms and C+D arms (depending on whether SS-PDS is used or not)

G. Compare cumulative incidence of surgical site infections and their types until Week 4 after surgery between A+C arms and B+D arms (depending on whether subcutaneous drain was used or not)

H. Compare incidence and cumulative incidence of wound dehiscence between A+C arms and B+D arms at Week 4 after surgery (depending on whether subcutaneous drain was used or not).

I. Compare incidence of adverse events, their type and relevance excluding incisional hernia, pains, surgical site infections, and wound dehiscence between A+B arms and C+D arms.

J. Create a surgical video of abdominal fascia closure using SS-PDS.

1. **Code Names (or Generic Names of Principal Components) of Investigational Product and Medical Device, Raw Material Quantities, Formulation and etc. (including** **comparator)**

**1) SS-PDS Experimental Group**

STRATAFIX Symmetric PDS Plus, suture size: 1/0, suture length: 45cm, needle size: 40mm (CT needle) (Ethicon, Somerville, NJ, USA)

**2) SS-PDS Control Group**

PDS Plus, suture size: 1/0, suture length: 90cm, needle size: 40mm (CT needle) (Ethicon, Somerville, NJ, USA)

**3) Subcutaneous Drainage Experimental Group**

Jackson-Pratt drain, 7 Fr (Manufacturer to be determined)

**4) Subcutaneous Drainage Control Group :** N/A

1. **Subject Eligibility and Exclusion Criteria, Target Sample Size and Calculation Rationale**
2. **Inclusion Criteria**

(1) Female patients planning to undergo elective midline laparotomy for gynecological diseases.

(2) Over 18 years old.

(3) ECOG performance status 0 - 2

1. **Exclusion Criteria**

(1) Patients with previous or present abdominal incisional hernia.

(2) Pregnant patients.

(3) Patients receiving external radiation treatment to the pelvis or abdomen.

(4) Patients allergic to PDS components.

(5) Patient with diseases that affect wound healing including uncontrolled diabetes, autoimmune vasculitis, and hepatocirrhosis.

(6) BMI>35

(7) Patients are excluded if they have used or are expected to use drugs that may affect wound healing such as bevacizumab. In case where bevacizumab is used, participation is possible if they have a four-week washout period before and after surgery, respectively.

(8) Patients who have had an abdominal midline laparotomy for the last 6 months.

(9) If patients had surgery for suspected infection.

1. **Target Sample Size and Calculation Rationale**

(1) Previous study results were referenced to assume 1-year cumulative incidence of incisional hernia as 0.13 in SS-PDS control group (C+D arm) [PMID: 26188742].

(2) No previous studies have investigated the incidence of incisional hernia after using barbed suture for abdominal fascia closure. Given that there are cases of very low incisional hernia incidence such as 1-2% in previous studies using conventional closure methods, 1-year cumulative incidence of incisional hernia in SS-PDS experimental group (A+B arm) is estimated to be 0.03.

(3) Alpha 0.05 (one-sided), power 0.8, drop-out rate 0.06, each arm (A+B vs C+D): 87 patients

(4) Therefore, the target sample size is 174.

1. **Recruitment Plan**

Investigators will recruit subjects for the study among the patients they come into contact while providing medical treatments. Investigators will not exclude patients who can participate in this study solely based on their ethnic or economic conditions. Investigators will make all efforts for patients who meet the study’s eligibility to join the study and to represent all the patients in the institution who undergo midline laparotomy due to gynecological diseases. Vulnerable population will not be included for this study.

1. **Trial Method**
2. **Schema**

**
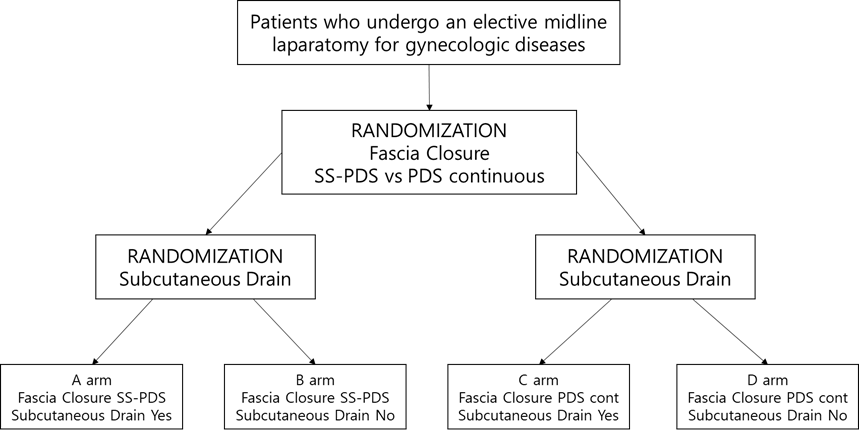
**

1. **Control Group Setting and Randomization**

(1) Acquisition of written consent

(2) Assignment of screening number.

A. Subjects will be given a screening number by each screening institution and institutions will make sure that the screening numbers are not overlapped.

B. The screening number will be in the form of ‘S_XX_01.’ XX is the initials of the screening institution.

| Screening institutions (random order) | Institution initials |
| --- | --- |
| Seoul Nat’l Univ. Bundang Hospital | SB |
| Samsung Medical Center | SM |
| National Cancer Center | NC |
| Severance Hospital | SV |
| Asan Medical Center | AM |
| Gachon Univ. Gil Medical Center | GG |
| Ajou Univ. Hospital | AJ |

(3) Eligibility check

A. Each institution will screen candidate subjects to judge whether they can participate in the study, fill out the eligibility check sheet, have it signed and stored.

B. Subjects who fail the screening will be treated as screening failures without moving to next steps. For successful screening results, scanned files of signed eligibility check sheets will be sent to KGOG.

(4) Eligibility review and randomization

A. KGOG will review the scanned files of signed eligibility check sheets sent from each institution and contact respective institution for any uncertainties or errors.

B. If subjects are confirmed eligible after KGOG reviews the scanned files of signed eligibility check sheets, randomization is not performed until one day before the surgery when the eligible subjects are hospitalized. Randomization is planned one day before the surgery in order to reduce dropout due to changed surgery plans.

C. If subjects are confirmed ineligible after KGOG reviews the scanned files of signed eligibility check sheets, each institution will be notified accordingly and the cases are treated as screening failure.

D. When each institution confirms that eligible subjects are hospitalized as planned and there is no change to the eligibility (change of surgery method, etc.), the institution shall notify KGOG accordingly and request randomization. KGOG will access the eCRF System (Redcap), input the data on the eligibility sheet and perform randomization. Random numbers will be assigned in the form of ‘R_XX_01’ where XX is the initials of the institutions.

D. Randomization table is created in advance and embedded in the eCRF system. The randomization table is created in advance by the Medical Research Cooperation Center of Seoul National University Bundang Hospital and will be imported to the eCRF system directly without the involvement of investigators. Details of the randomization table are as follows:

A) The ratio of SS-PDS experimental group vs. control group is 1:1

B) The ratio of subcutaneous drainage experimental group vs. control group is 1:1

C) Stratification factors are institutions and BMI (≤30 vs >30), Surgery indication (Confirmed or suspected cancer vs not). The Stratification factors are applied to all of A), B), and random.

D) Block randomization

(5) Notification of randomization results

A. Randomization results should not be notified until just before the fascia closure begins during surgery.

B. Just before the fascia closure begins, each institution will notify the screening number to KGOG by phone and check the randomization result by phone as well.

C. Randomization results will not be notified using numbers such as 0 or 1 but in the form of ‘SS-PDS experimental group’ or ‘subcutaneous drain control group.’ After being notified of the randomization results, each institution will say out the result again to KGOG so as to avoid any error.

D. KGOG will create a record on the eCRF according to random number and inform the pair of screening number-randomization number to each institution so that the institution can input data on the eCRF.

(6) Nondisclosure of randomization results

A. SS-PDS randomization results

A) Study subjects are not notified of the randomization results.

B) In order to minimize investigator influence on the randomization at the time of incisional hernia diagnosis, randomization results are not recoded on medical records but only on CRF.

B. Subcutaneous drain randomization results

Randomization results are disclosed

(7) Undisclosed randomization results are disclosed to:

A. investigators: investigators at each institution can view CRF and check randomization results without a separate approval procedure.

B. study subjects: if investigators see it necessary for medical purposes, they can inform the subjects of the randomization results after approval by the principal investigator.

(8) Interim Analysis

A. Interim analysis for the initial safety review

A) When the 10th subject is allocated to A+B arm and finishes his/her Week 4 visit, an evaluation will be performed on 10 subjects in A+B arm for incidence, types, and grades of surgical site infections, wound dehiscence, and other adverse events.

B) Evaluation results will be reported to IRB. If incidence, types, and grades of adverse events are judged to exceed the usual range, possible measures including trial discontinuation will be considered.

B. Interim analysis for wound dehiscence of subcutaneous drain

A) An evaluation will be performed when the 44th subject of A+C arm and B+D arm, respectively, completes his/her week 4 visit.

B) If wound dehiscence incidence of A+C arm is lower than B+D arm (one-sided p < 0.005), it will be decided that subcutaneous drain insertion reduces wound dehiscence and further randomization will be stopped. That is to say that subcutaneous drain will be inserted to all subject from then.

1. **Investigational Product Dosage, Application/Administration Methods, Combination Therapy, and Reasons for Comparator (as applicable)**

(1) SS-PDS arm (fascia closure using SS-PDS)

A. Instructions

A) STRATAFIX Symmetric PDS Plus, suture size: 1/0, suture length: 45cm, needle size: 40mm (CT needle) (Ethicon, Somerville, NJ, USA)

B) Running suture will be used as recommended by the manufacturer and stated in previous studies [PMID: 28603661] (<https://www.youtube.com/watch?v=PTYtYvSaGMg>, <https://youtu.be/TPyulS4fQgQ>)

C) Peritoneum closure should be performed. If peritoneum closure is impossible due to peritoneotomy, it may not be performed.

D) For fascia closure, stiches will be made 5-8 mm apart including 5-8 mm thick tissues on both sides of the incision surface.

E) It is allowed to make additional stiches for fascia closure with a few interrupted sutures using PDS or other sutures based on the investigator’s judgment.

F) Suture length is recommended to be 4 times longer than the skin incision. That is, if the skin incision is 20cm, it is recommended to use 80cm or longer suture for closure.

G) Cares should be made to stitch only the aponeurosis located at the ventral rectus muscle and not to stitch too much fat tissues or muscles. It is recommended to include dorsal sheath of the rectus muscle in the stiches from the cephalic side of the arcuate line, if possible.

H) It is recommended to start the stiches from cephalic end and caudal end of fascia incision. That is, the stiches should not be ended at the caudal end (fascia touching the public bone).

I) If the stiches end in the middle of the incision, additional stiches should be made 2-3 times in reverse direction as recommended and finished. Also, there should be an overlap of 2cm or longer with the next suture.

F) Stitching can start from one side of the incision wound if necessary (Appendix Figure 1)

G) Since barbed suture does not become loose after stitching unlike general suture, the suture should not be pulled hard for closure. Also, constant force should be applied for the closure so that the pressure is evenly distributed over the stitches.

B. Reasons for selection

The reasons are stated under the section ‘Background.’

(2) SS-PDS control group (fascia closure using conventional sutures instead of barbed suture)

A. Instructions

A) PDS Plus, suture size: 1/0, suture length: 90cm, needle size: 40mm (CT needle) (Ethicon, Somerville, NJ, USA)

B) Running suture will be used as stated in previous studies [PMID: 26188742]

C) Peritoneum closure should be performed. If peritoneum closure is impossible due to peritoneotomy, it may not be performed.

D) For fascia closure, stiches will be made 5-8 mm apart including 5-8 mm thick tissues on both sides of the incision surface.

E) It is allowed to make additional stiches for fascia closure with a few interrupted sutures using PDS or other sutures based on the investigator’s judgment.

F) Suture length is recommended to be 4 times longer than the skin incision. That is, if the skin incision is 20cm, it is recommended to use 80cm or longer suture for closure.

G) Cares should be made to stitch only the aponeurosis located at the ventral rectus muscle and not to stitch too much fat tissues or muscles. It is recommended to include dorsal sheath of the rectus muscle in the stiches from the cephalic side of the arcuate line, if possible.

H) Stitches can start from both ends or one end of fascia incision. In case of using 2 sutures, each suture should be fixed with a knot, respectively, and stitches should be overlapped for more than 2cm.

B. Reasons for selection

A) Fascia closure of abdominal midline incision has been developed from interrupted sutures to continuous running sutures using monofilament sutures. In recent randomization studies, suture techniques that include less tissues were found to reduce the incisional hernia incidence. [PMID: 20395846,12594682, 26188742, 19917943].

B) The European Hernia Society recommends using sutures with delayed absorption, and this is accepted as a standard [PMID: 25618025].

C) 2 meta-analyses compared sutures with delayed absorption were compared and non-absorbent sutures. While sutures with delayed absorption had similar incisional hernia with non-absorbent sutures, suture sinus and wound pain were reduced. Use of sutures with delayed absorption is recommended for this reason [PMID: 20395846, 22061310].

D) When sutures with delayed absorption were compared to sutures with rapid absorption in a recent meta-analysis, less incisional hernia was associated with sutures with delayed absorption [PMID: 20395846]

(3) Subcutaneous drain experimental group (subcutaneous drain is inserted)

A. Instructions

A) Device: Jackson-Pratt drain, 7 Fr (manufacturer to be determined)

B) Place closed, negative drain at hypodermis. The drain should be inserted through a skin perforation separate from the surgical incision.

C) Subcutaneous tissue closure is not necessary, but can be performed based on the investigator’s judgment.

D) Skin closure can be performed using stapler or non-absorbent sutures whichever is a general method of each institution.

E) Based on the investigator’s judgment, the subcutaneous drain can be removed any time between 3 days and 14 days after operation when the drainage volume is less than 10cc. If daily drainage volume is greater than 10cc until when the applicable patient is discharged, the subcutaneous drain can be removed based on the investigator’s judgment. If daily drainage volume is greater than 10cc when 14 days have passed after surgery, the subcutaneous drain can be maintained or removed based on the investigator’s judgment.

F) In case it is impossible to insert subcutaneous drain because the subcutaneous layer is too thin, the drain may not be inserted based on the investigator’s judgment.

B. Reasons for selection

The reasons are stated under the section ‘Background.’

(4) Subcutaneous control group (subcutaneous drain is not inserted)

A. Instructions

1. Subcutaneous drain is not inserted.

2. Subcutaneous tissue closure is not necessary, but can be performed based on the investigator’s judgment.

3. Skin closure can be performed using stapler or non-absorbent sutures whichever is a general method of each institution.

B. Reasons for the choice

The reasons are stated under the section ‘Background.’

1. **Observation Items, Clinical Test Items, Observation and Test Methods**

| Observation Items | Observation and Test Methods |
| --- | --- |
| 1-year cumulative incidence of incisional hernia after surgery | Subjects are advised to make outpatient visits for Year 1 after surgery. Visits made between 9 and 15 months after surgery can be counted as Year 1 visit. Investigators will conduct a physical checkup to diagnose incisional hernia. Investigators can refer to post-surgery image test result. If investigators are not sure of incisional hernia, a diagnostic test can be carried out. Subjects will be censored if they underwent midline laparotomy once again after receiving the surgery from this study, are dead or lost to follow-up at the time of event. Investigators will not be reminded of group allocation and will be blinded until Year 1 visit is completed. |
| 4-week cumulative incidence and types of surgical site infection after surgery | Subjects are advised to make outpatient visits for Week 4 after surgery. Visits made between 3 to 5 weeks after surgery can be counted as Week 4 visit. Investigators will conduct a physical checkup to diagnose surgical site infection. If subjects make outpatient visits before Week 4 after surgery, investigators should check for surgical site infections and their types. All information obtained from visits made immediately after surgery up to Week 4 will be used to diagnose all surgical site infections. CDC standards will be used to determine surgical site infections and their types. (https://www.cdc.gov/nhsn/PDFs/pscManual/17pscNosInfDef_current.pdf). If any surgical site infection is observed according to CDC standards, the patient will be judged to have a surgical site infection. Investigators will not be reminded of group allocation. |
| 4-week cumulative incidence of wound dehiscence after surgery | Subjects are advised to make outpatient visits for Week 4 after surgery. Visits made between 3 to 5 weeks after surgery can be counted as Week 4 visit. Investigators will conduct a physical checkup to diagnose wound dehiscence. Wound dehiscence is defined as all wound dehiscence in any depth and length. If subjects make outpatient visits before Week 4 after surgery, investigators should check for wound dehiscence. All information obtained from visits made immediately after surgery up to Week 4 will be used to calculate all cumulative incidence. Events that will be included in the calculation of cumulative incidence will be: when wound dehiscence is observed even just once until Week 4 after surgery; when removal of stapler or suture is impossible at Week 4; or wound dehiscence is observed during follow-up after stapler or suture was removed at Week 4. |
| Wound dehiscence incidence at week 4 after surgery | Subjects are advised to make outpatient visits for Week 4 after surgery. Visits made between 3 to 5 weeks after surgery can be counted as Week 4 visit. Investigators will conduct a physical checkup to diagnose wound dehiscence. Wound dehiscence at Week 4 is defined as: when a wound is expanded at Week 4 evaluation; when removal of stapler or suture is impossible; or wound dehiscence is observed during follow-up after stapler or suture was removed at Week 4. If wound dehiscence was observed before Week 4 but it disappeared at Week 4, the subject is not counted to have wound dehiscence at Week 4 after surgery. When stapler or suture is removed at Week 4 visit, a follow-up visit or phone counseling is required. If no wound dehiscence is discovered from the follow-up visit or phone counseling, the subject is not counted to have wound dehiscence. However, if wound dehiscence is discovered from the follow-up visit or phone counseling, the subject is counted to have wound dehiscence. |
| BPI-K Score | BPI-Korean version is used at baseline, Day 2 and Day 4 after surgery. |
| NRS Pain Score | Pain scores, measured by NRS which is a usual way of monitoring surgical patients, and record times are collected from immediately after surgery until 10pm of Day 4 after surgery. |
| Demographics of subjects, surgical information | Date of birth, height, weight, current smoker, medical history, pre- and postoperative chemotherapy, corticosteroids, previous abdominal surgery, ASA classification, type of surgery, suture length, wound length, operation time, antibiotic prophylaxis, intra-abdominal drain, subcutaneous drain, DVT prophylaxis, Patient controlled analgesia (Y/N, type IV or epidural), wound anesthetics infiltration system, perioperative complications, transfusion, postoperative radiation, ICU care |
| Incidence, types, grades and relevance of adverse events excluding incisional hernia, pains, surgical site infections, and wound dehiscence | Adverse events are collected by listening to the subject’s medical history and conducting a physical checkup during hospitalization for surgery, Week 4 and Year 1 visits after surgery. All visits and medical records until Year 1 after surgery will be used. CTCAE ver 4.0 will be used to grade each adverse event. |

1. **Efficacy Evaluation Standards, Evaluation Methods**

| Purpose | Analysis and Evaluation Methods | Cohorts for analysis |
| --- | --- | --- |
| Compare cumulative incisional hernia incidence until Year 1 after surgery between A+B arm and C+D arm. | Cumulative incidence of incisional hernia of A+B arm will be compared with that of C+D arm using chi-square or Fisher's exact test. P value 0.05 will be used to determine significance. | All patients who undergo surgery. Patients who do not have incisional hernia will be censored at last visit. Incidence will be estimated from time-occurrence curve. |
| Compare incisional hernia incidence curve by time between A+B arm and C+D arm. | Time-occurrence curve of incisional hernia will be depicted using Kaplan-Meier method and be compared between A+B vs C+D arm using log-rank test. P value 0.05 will be used to determine significance. | All patients who undergo surgery |
| Compare cumulative incidence of surgical site infections and their types until Week 4 after surgery between A+B arm and C+D arm. | Cumulative incidence of SSI of A+B arm will be compared with that of C+D arm using chi-square or Fisher's exact test. P value 0.05 will be used to determine significance. Type of SSI will be summarized. | Patients who undergo surgery |
| Compare cumulative wound dehiscence incidence at Week 4 after surgery between A+B arm and C+D arm. | Cumulative incidence of wound dehiscence of A+B arm will be compared with that of C+D arm using chi-square or Fisher's exact test. P value 0.05 will be used to determine significance. | Patients who undergo surgery and complete week 4 visit |
| Compare wound dehiscence incidence at Week 4 after surgery between A+B arm and C+D arm. | Incidence of wound dehiscence of A+B arm will be compared with that of C+D arm using chi-square or Fisher's exact test. P value 0.05 will be used to determine significance. | Patients who undergo surgery and complete week 4 visit |
| Compare BPI-K scores that are obtained at baseline, Day 2 and Day 4 after surgery between A+B arm and C+D arm. | BPI score (total and each question) obtained at baseline will be compared between A+B vs C+D arm. BPI score (total and each question) obtained at postoperative day 2 will be compared between A+B vs C+D arm. BPI score (total and each question) obtained at postoperative day 4 will be compared between A+B vs C+D arm. Student t-test will be used. P value 0.05 will be used to determine significance. If BPI score at baseline is imbalanced between arms, change of BPI score (from baseline to postoperative day 2, 4) will be compared. | Patient who undergo surgery and complete BPI |
| Compare NRS pain scores collected until Day 4 after surgery between A+B arm and C+D arm. | NRS collected for postoperative 4 days will be compared between A+B vs C+D arm using linear mixed model. P value 0.05 will be used to determine significance. | Patients who undergo surgery and NRS are measured |
| Compare cumulative incidence and types of surgical site infections until Day 4 after surgery between A+C arm and B+D arm (depending on whether subcutaneous drain is inserted or not) | Cumulative incidence of SSI of A+C arm will be compared with that of B+D arm using chi-square or Fisher's exact test. P value 0.05 will be used to determine significance. Type of SSI will be summarized. | Patients who undergo surgery |
| Compare cumulative wound dehiscence incidence at Week 4 after surgery between A+C arm and B+D arm (depending on whether subcutaneous drain is inserted or not) | Cumulative incidence of wound dehiscence of A+C arm will be compared with that of B+D arm using chi-square or Fisher's exact test. P value 0.05 will be used to determine significance. | Patients who undergo surgery and complete week 4 visit |
| Compare wound dehiscence incidence at Week 4 between A+C arm and B+D arm (depending on whether subcutaneous drain is inserted or not) | Incidence of wound dehiscence of A+C arm will be compared with that of B+D arm using chi-square or Fisher's exact test. P value 0.05 will be used to determine significance. | Patients who undergo surgery and complete week 4 visit |
| Incidence, types, grades and relevance of adverse events excluding incisional hernia, pains, surgical site infections, and wound dehiscence. | Incidence, type and grade of adverse events except incisional hernia, pain, SSI, wound dehiscence will be summarized using appropriate metrics. They will be compared using appropriate statistical methods. | Patients who undergo surgery. |

1. **Distinctions from conventional treatments and studies**

(1) Using barbed suture for abdominal fascial closure falls within the range of treatments approved by US FDA and Korea MFDS.

(2) Using barbed suture for abdominal fascial closure has a possibility to reduce potential incisional hernia.

(3) There is a lack of systematical studies for abdominal fascial closures using barbed suture.

1. **Benefits for and Risks to Subjects**

(1) Predictable side effects/risks and countermeasures

A. Regardless of predictable side effects/risks – causality,

this trial targets female patients who undergo midline laparotomy. Therefore, various adverse events involved with surgery may be observed. Also, a considerable number of subjects are expected to be diagnosed as and treated for malignant tumor, and adverse events may be observed in this regard.

A) Blood and lymphatic system disorders: anemia, febrile neutropenia, hemolysis, leukocytosis

B) Cardiac disorders: palpitations, sinus bradycardia

C) Ear and labyrinth disorders: vertigo

D) Eye disorders: Blurred vision, conjunctivitis, dry eye

E) Gastrointestinal disorders: abdominal distension, abdominal pain, anal pain, ascites, bloating, constipation, diarrhea, dry mouth, dyspepsia, enterocolitis, fecal incontinence, flatulence, ileus, intra-abdominal hemorrhage, nausea, vomiting

F) General disorders and administration site conditions: chills, edema face, edema limb, fatigue, fever, infusion related reaction, infusion site extravasation, irritability, localized edema, non-cardiac chest pain, pain

G) Infections and infestations: abdominal infection, bladder infection, catheter related infection, peritoneal infection, pharyngitis, stoma site infection, urinary tract infection, wound infection

H) Injury, poisoning and procedural complications: intraoperative gastrointestinal injury, intraoperative venous injury, intraoperative urinary injury, large intestinal anastomotic leak, postoperative hemorrhage, wound complication, wound dehiscence

I) Investigations: activated partial thromboplastin time prolonged, alanine aminotransferase increased, alkaline phosphatase increased, aspartate aminotransferase increased, platelet count decreased, urine output decreased, weight gain, weight loss

J) Metabolism and nutrition disorders: anorexia, dehydration

K) Musculoskeletal and connective tissue disorders: back pain, flank pain, myalgia, neck pain

L) Psychiatric disorders: anxiety, delirium, insomnia

M) Renal and urinary disorders: hematuria, proteinuria, urinary frequency, urinary incontinence, urinary retention, urinary urgency

N) Reproductive system and breast disorders: menorrhagia, irregular menstruation, pelvic pain, vaginal discharge, vaginal dryness, vaginal hemorrhage

O) Respiratory, thoracic and mediastinal disorders: atelectasis, cough, dyspnea, hypoxia, pleural effusion, sore throat

P) Skin and subcutaneous tissue disorders: alopecia, pruritus, purpura, urticarial

Q) Vascular disorders: flushing, hot flashes, phlebitis, thromboembolic event

B. Side effects/risks from using barbed suture and countermeasures

A) Side effects/risks

Barbed suture has been used for various soft-tissue closures since years ago. SS-PDS is approved to be used for abdominal fascia closure. However, there are little clinical experience of using SS-PDS for abdominal fascia closure. Potential side effects/risks would include increased incisional hernia, surgical site infections, wound dehiscence and pain but increase is not expected.

B) Countermeasures

When the 10th subject is allocated to the A+B arm and completes his/her week 4 visit, an evaluation will be performed on 10 subjects in A+B arm for surgical site infections, wound dehiscence and incidence, types and grades of other adverse events. Evaluation results will be reported to IRB. If incidence, types, and grades of adverse events are judged to exceed the usual range, possible measures including trial discontinuation will be considered.

C. Side effects/risks from using subcutaneous drain and countermeasures

Use of subcutaneous can be regarded as one of standard techniques. Therefore, it is judged that there will be no additional side effects/risks compared to standard treatments.

(2) Statement of rationale (or expected scientific/medical benefits) for continuing the trial despite predictable side effects/risks

It is judged that expected benefits from the trial (incisional hernia can be reduced by using SS-PDS for abdominal fascia closure) outweigh potential side effects/risks (increased incisional hernia, surgical site infections, wound dehiscence, and pains in SS-PDF arm) from the trial.

1. **Discontinuation Criteria and Dropouts from Study**

**(1) Discontinuation**

A. The principal investigator can stop the study if it is judged that SS-PDS’s safety is low. Discontinuation of the trial can be considered when serious adverse events occurred from the interim analysis results.

B. The principal investigator can stop the trial if recommended by IRB.

C. The principal investigator can stop the trial if it is impossible to carry out the trial properly.

**(2) Removal of subjects from study**

A. Subjects can remove from study unconditionally while they are participating in the study.

B. Subjects are removed if they do not undergo midline laparotomy. No follow-up will be performed afterwards.

C. If incisional hernia is diagnosed in 4 weeks after surgery, no follow-up will be performed afterwards. In this case, subjects are considered to have completed the trial participation. If incisional hernia is diagnosed when 4 weeks have not elapsed after surgery, follow-up observations will be made until Week 4 after surgery.

D. If subjects undergo another abdominal midline laparotomy after surgery, the subject will be withdrawn from the trial. No follow-up will be performed afterwards.

E. If it is judged that a subject is no longer able to participate in the trial due to the subject’s noncooperation, the principal can decide to discontinue the subject’s participation.

F. If a subject is withdrawn from the trial, data collected until the time of withdrawal are used for the trial.

1. **Evaluation Standards, Methods and Reporting of Safety Including Side Effects**

**(1) Causality Evaluation**

A. Investigators will determine that there is or there is no causal relationship with the SS-PDS use (including control group’s suture). If it is difficult for the investigator to make a judgment, it will be judged to have a causal relationship.

B. Causality will not be evaluated for the use of subcutaneous drain.

**(2) Evaluation Methods**

A. Compare the entire incidence of adverse events that have causal relationship with the SS-PDS use (including control group’s suture) between A+C arm and B+D arm. (significance is judged by p-value 0.05).

B. Compare the incidence of adverse events by type that have causal relationship with the SS-PDS use (including control group’s suture) between A+C arm and B+D arm (significance is judged by p-value 0.05).

C. Compare the entire incidence of grade 3 or above adverse events that have causal relationship with the SS-PDS use (including control group’s suture) between A+C arm and B+D arm (significance is judged by p-value 0.05).

D. Compare the incidence grade 3 or above adverse events by type that have causal relationship with the SS-PDS use (including control group’s suture) between A+C arm and B+D arm (significance is judged by p-value 0.05)

**(3) Reporting Methods**

A. Adverse events from SS-PDS arm that are serious, unpredicted and in causal relationship (SS-PDS SUSAR) : Investigators will take below measures in 7 days from the day they became aware of such adverse events.

A) Report to IRB of the applicable institution;

B) Report to the principal investigator: KGOG which is delegated by the principal investigator will report to MFDS in 7 days from the day such adverse events were reported and deliver the information to other institutions so that such adverse events are reported to IRBs the institutions, respectively.

C) However, such adverse events should be directly reported to the principal investigator events if they lead to death or are life-threatening.

B. Adverse events that are not SS-PDS SUSAR

A) KGOG which is delegated by the principal investigator will collect all adverse events from all participating institutions once a year and report to IRBs of all the participating institutions.

B) Adverse events that are not SS-PDS SUSAR can also be reported to IRB individually based on the investigator’s judgment. In this case, the adverse events should also be notified to the principal investigator. The principal investigator will review the adverse events and take necessary measures including reporting to MFDS and IRBs of other institutions.

1. **Data & Safety Monitoring Plans (DSMP)**

(1) Monitoring Plan

A. Monitoring staff: CRAs of Korean Gynecologic Oncology Group (KGOG)

B. On line monitoring: Monitoring staff will review the data uploaded on the eCRF once a month and send out queries to each institution for any uncertain or erroneous data. The staff will also make requests to the institutions for cases with delayed input.

C. Face-to-face monitoring: Visits will be made when there are 2 subjects and 10 subjects who completed Week 4 visits for each institution and perform monitoring activities according to KGOG monitoring SOP.

(2) Monitoring Frequency

On line monitoring will be conducted once a months and face-to-face monitoring will be conducted 2 times during the trial period for each institution.

(3) Monitoring Result Reporting

A. KGOG will draft monitoring result reports after conducting on line monitoring (monthly) and face-to-face monitoring activities and repot to the principal investigator.

B. The principal investigator will send the report to responsible investigators of applicable institutions and request the investigator to report to IRB.

C. For any matters that require a separate reporting to IRB such as non-compliance, the principal investigator will request the responsible investigator of the institution to report to IRB.

D. The principal investigator can request a corrective plan to each institution’s responsible investigator depending on the contents included in the result report.

1. **Data Analysis and Statistical Analysis Methods**

(1) Information on inter-group univariate comparative analysis is stated under the sections ‘Efficacy Evaluation Standards, Evaluation Methods’ and ‘Evaluation Standards, Methods and Reporting of Safety Including Side Effects.’

(2) In case of inter-group differences for the subjects and surgical information factors, a multivariate comparative analysis will be conducted including the different factors. For example, if there is age difference between A+B arm and C+D arm, a multivariate analysis will be conducted to include the age factor in addition to the univariate comparative analysis.

1. **Trial Schedule**

Enrolment rate: 9/month

Submission date to health authority / ethics: March, 2020

Start of subject enrolment: August, 2020

End of Subject enrolment: March, 2022

End of Study: May, 2023

Report (as described in the contract, e.g. draft publication): Dec, 2023

Planned publication /presentation: Dec, 2023

1. **Measures for Safety and Protection of Subjects**
2. **Basic considerations to ensure ethical conduct of the trial**

The trial will be in compliance with the Declaration of Helsinki (revised in 2013) and ICH-GCP.

The trial will be conducted after obtaining IRB approval.

1. **Process for subjects to express consent**

(1) Investigators who will explain to the subjects and obtain their consent: Ki-dong Kim, Yong-beom Kim, Jae-hong Noh, Dong-hoon Seo, Joo-hyeon Kim, Joo-young Kim, Woo-yeon Hwang

(2) Persons who will express their consent: subjects

(3) Waiting period between trial explanation and consent acquisition: 5 minutes

(4) Measures to minimize the possibility of coercion or negative influence: subjects will be explained clearly that there will be no disadvantages even if they do not participate in the trial.

(5) Language to be used by investigators in the process of trial explanation and consent acquisition: Korean

(6) Language that can be understood by the subjects or their representatives: Korean

(7) Information and the consent form provided to the subjects or their representatives: to be submitted as separate documents

1. **Compensations for the subjects**

(1) 30,000 won will be paid out as travel expense for the subjects on their Week 4 visit and Year 1 visit after surgery.

(2) If subjects are withdrawn from the trial, travel expense until the withdrawal will be paid out.

(3) Subject will not be paid for travel expense at baseline visit and hospitalization for surgery because these visits are considered to be related to the subjects’ surgery.

1. **Private information protection of the subjects**

(1) Patient medical record numbers and pathology numbers will be managed in a separate file under the responsibility of the principal investigator which will be coded so that one’s personal information cannot be identified from the trial data.

(2) Trial data is stored on the eCRF which is accessible only with a password.

(3) In accordance with Article 15 of the Enforcement Regulations of the Bioethics and Safety Act, trial-related records should be kept for 3 years from the point the trial is completed. Documents with expired storage period will be shredded according to Article 16 of the Enforcement Decree of the Personal Information Protection Act.

1. **Additional protection measures in case vulnerable population is included**

Vulnerable population is not included in the trial.

1. **Disposal of human body materials**

No human body materials are collected.

1. **References**

Inserted as Pubmed ID in the body of the protocol.

1. **Creating a surgical video for abdominal fascia closure operating using SS-PDS**

1) Subjects can express consent for creating a video optionally.

2) A video will be produced for the abdominal fascia closure technique using SS-PDS for subjects who expressed consent in advance. Any personal information or sensitive body parts should not be included in the video.

3) Even if a subject expressed consent to create a video, he/she may not agree to produce the video later.

4) When a video is produced, the subject will be notified of the production immediately after the surgery. If the subject wants to watch the video, the investigator should cooperate.

**Appendix Figure 1**


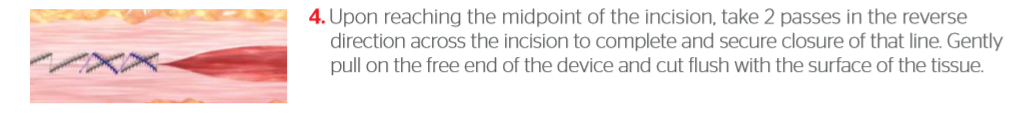


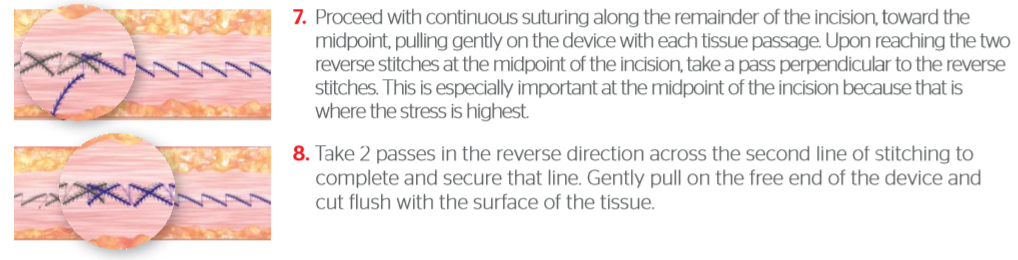


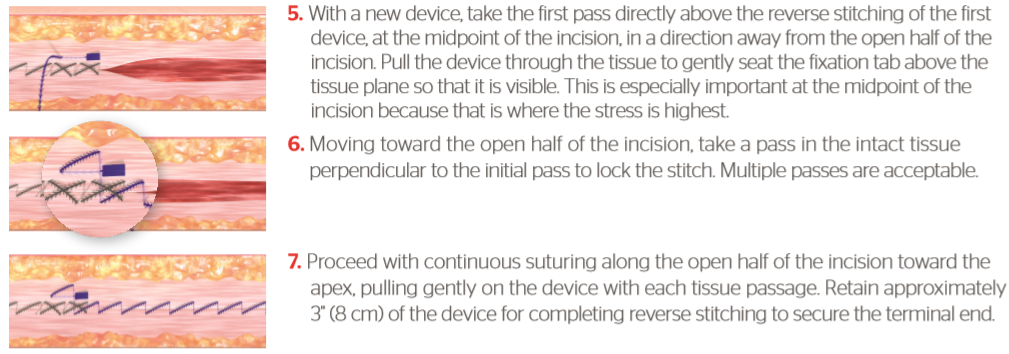

Supplement: S5 File — (DOCX) [file pone.0337036.s005.docx]
