# Supplementary material for: Effect of fascial closure using barbed sutures on incisional hernias in midline laparotomy for gynecological diseases: A multicenter randomized controlled trial (KGOG 4001)
Source: PLoS One. 2025 Nov 19;20(11):e0337036. doi: 10.1371/journal.pone.0337036 (PMC12629448; doi:10.1371/journal.pone.0337036)

**부인과질환으로 정중절개 개복술을 받는 여성에서 미늘봉합사를 이용한 근막봉합이 절개창탈장 발생에 미치는 영향**

**Effect of Barbed Suture Fascia Closure on Incisional hernia in Midline Laparotomy for Gynecological Diseases (BARBHER)**

**Version No: 0.4**

**책임연구자 소속: 분당서울대학교병원**

**책임연구자 이름: 김기동**

**연구 개요**

| 연구제목 | (국문) 부인과질환으로 정중절개 개복술을 받는 여성에서 미늘봉합사를 이용한 근막봉합이 절개창탈장 발생에 미치는 영향 |
| --- | --- |
|  | (영문) Effect of Barbed Suture Fascia Closure on Incisional hernia in Midline Laparotomy for Gynecological Diseases (BARBHER) |
| 책임연구자 | 산부인과 김기동교수 |
| 연구비 지원기관 | 존슨앤존슨 |

| 연구 목적 | 부인과질환으로 정중절개 개복술을 받는 여성에서 미늘봉합사를 이용한 근막봉합이 절개창탈장 발생을 감소시키는지 알아봄 |
| --- | --- |
| 연구 설계 | 전향적, 다기관, 무작위배정 임상시험 |
| 연구 기간 | IRB승인일로부터 40 개월 |
| 연구 대상 | 부인과질환으로 정중절개 개복술을 받는 여성 |
| 연구 대상자 수 | 174명 |
| 취약한 연구대상자 | 취약한 연구대상자는 참여하지 않음 |
| 시험약/의료기기 | STRATAFIX Symmetric PDS Plus (SS-PDS), suture size: 1/0, suture length: 45cm, needle size: 40mm (CT needle) (Ethicon, Somerville, NJ, USA) |
| 용법 및 용량 | 제조사에서 제공하는 사용법에 따라 근막봉합 |
| 연구 방법 | 1:1 의 비율로 SS-PDS 실험군과 대조군으로 무작위배정  SS-PDS 실험군: 미늘봉합사 (SS-PDS) 를 사용하여 근막봉합  SS-PDS 대조군: 미늘봉합사가 아닌 통상적인 봉합사를 사용하여 근막봉합  1년 절개창탈장 누적발생률을 실험군 vs 대조군 비교  이외, 수술부위감염, 상처벌어짐, 통증, adverse event 를 비교  또한, 2차 목적으로 피하드레인 여부에 따른 무작위배정과 그에 따른 비교 시행 |
| 주요 선정기준 | 1. 부인과질환으로 정중절개 개복술을 받을 예정인 여성  2. 나이 18세 초과  3. ECOG performance status 0 - 2 |
| 주요 선정  제외기준 | 1. 복부 절개창탈장이 이전에 있었거나 현재 있음  2. 임신 중  3. 골반 혹은 복부에 외부 방사선치료를 받은 경우  4. PDS 성분에 알레르기가 있는 경우  5. 조절되지 않는 당뇨, 자가면역 혈관염, 간경화 등 상처치유에 영향을 줄 수 있는 질환  6. BMI>35  7. 베바시주맙과 같이 상처치유에 영향을 줄 수 있는 약물을 사용하였거나 사용예정인 경우. 베바시주맙을 사용하는 경우 수술 전과 후 각 4주의 휴약기를 가지면 참여 가능함  8. 최근 6개월 이내 복부 정중절개 수술을 받은 경우  9. 감염이 의심되어 수술을 시행하는 경우 |
| 유효성 평가 | SS-PDS 실험군의 1년 절개창탈장 누적발생률이 SS-PDS 대조군에 비해 낮으면 유효하다고 판정 (p value 0.05 기준) |
| 안전성 평가 | Adverse events 의 빈도와 grade 를 SS-PDS 실험군, 대조군 간 비교 |
| 검사/방문일정 | Baseline: eligibility check  수술: SS-PDS 혹은 통상적 봉합사로 근막봉합 시행, 피하드레인 삽입, 수술 관련 변수 수집, 수술 후 통증 조사  수술 후 4주: 수술부위감염, 수술벌어짐 조사  수술 후 1년: 절개창탈장 여부 판정 |
| 통계적 분석방법 | SS-PDS 실험군, 대조군 간 절개창탈장 발생율 비교. Chi-square or Fisher’s exact test 사용 |
| 기대효과 및  예상결과 | 표준 수술방법이 미늘봉합사를 사용한 근막봉합으로 변경  절개창탈장을 감소시킴으로서 수술 환자의 삶의 질 향상, 의료 비용 절감 |

**연구계획서**

1. **연구 제목**

부인과질환으로 정중절개 개복술을 받는 여성에서 미늘봉합사를 이용한 근막봉합이 절개창탈장 발생에 미치는 영향

1. **연구의 실시기관 명칭 및 주소, 기관 별 책임연구자**

| 실시기관 명칭 (무순) | 주소 | 책임연구자 |
| --- | --- | --- |
| 분당서울대학교병원 | 경기도 성남시 분당구 구미로 173번길 82 | 김기동 (산부인과) |
| 삼성서울병원 | 서울특별시 강남구 일원로 81 | 최철훈 (산부인과) |
| 국립암센터 | 경기도 고양시 일산동구 일산로 323 | 임명철 (자궁난소암센터) |
| 세브란스병원 | 서울특별시 서대문구 연세로 50-1 | 이정윤 (산부인과) |
| 서울아산병원 | 서울특별시 송파구 올림픽로 43길 88 | 박정열 (산부인과) |
| 가천대 길병원 | 인천광역시 남동구 남동대로 774번길 21 | 이광범 (산부인과) |
| 아주대학교병원 | 경기도 수원시 영통구 월드컵로 164 | 장석준 (산부인과) |

*분당서울대학교병원 김기동 교수가 coordinating 책임연구자

1. **연구책임자 및 공동연구자 성명 및 직명**
2. **연구책임자**

김기동 (산부인과 부교수)

1. **공동연구자**

김용범 (산부인과 교수)

노재홍 (산부인과 부교수)

서동훈 (산부인과 부교수)

김주현 (산부인과 진료교수)

김주영 (산부인과 전임의)

황우연 (산부인과 전임의)

1. **연구담당자**

오유민 (산부인과 연구간호사)

1. **임상시험용 의약품 관리약사 / 임상시험용 의료기기 관리자**

분당서울대학교병원 의료기기연구개발센터

1. **연구 의뢰기관
   1) 연구 의뢰기관 명칭 및 주소:** 의뢰기관 없음
   **2) 모니터요원 성명 및 직명:** 대한부인종양연구회 (KGOG) CRA
2. **연구비 지원기관 명칭 및 주소**
3. **명칭:** 존슨앤존슨
4. **주소:** New Brunswick, New Jersey
5. **RFP ID Number**: 2019-SFX-GEN-02
6. **예상연구기간**

IRB승인일로부터 40 개월

1. **연구 대상 질환**

정중절개 개복술을 요하는 부인과질환

1. **연구의 배경 및 목적**
2. **연구 배경**

절개창탈장은 수술절개부위에 발생한 탈장임 [PMID: 19495920]. 절개창탈장은 복부수술의 흔한 합병증으로 10-23% 정도의 발생률을 보이며, 고령, 비만, 대사증후군, 만성폐쇄성호흡기질환, 영양불량, 복부혈관류 등 고위험군에서는 38% 까지도 발생하는 것으로 알려짐 [PMID: 21254041]. 절개창탈장 발생에 영향을 미치는 수술인자로는 절개의 종류와 크기, 복벽봉합의 방법 등이 있음 [PMID:10195729, 3159324, 3954314, 2804595]. 절개창탈장은 삶의질을 저하시키고 많은 비용을 발생시킴 [PMID:26206646].

많은 리뷰와 가이드라인들이 적절한 복부 근막봉합의 방법을 제시하고 있음 [PMID10714638, 15796944, 12594682, 11379640, 9926810, 20395846]. 메타분석에 따르면 비연속봉합 (interrupted suture) 에 비해 늦게 흡수되는 모노필라멘트 봉합사를 사용한 연속봉합 (running suture) 이 절개창탈장의 발생을 감소시켰음 [PMID: 20395846,12594682, 26188742, 19917943]. 그러나, 적절한 봉합사와 봉합방법을 사용하더라도 여전히 10% 가 넘는 환자들이 복부수술 후 절개창탈장을 경험함 [PMID: 26188742]. 따라서, 복부 근막봉합의 방법을 개선시키는 것이 필요함.

미늘봉합사는 표면에 미늘이 있는 봉합사임. 여러 상황에서 미늘봉합사는 미늘이 없는 봉합사와 유사하거나 더 좋은 봉합결과를 보임 [PMID: 28603661]. STRATAFIX Symmetric PDS Plus (SS-PDS) 는 근막같이 압력이 높은 부위 봉합에 사용할 수 있는 미늘봉합사임. SS-PDS 는 강도와 조직잡는능력에서 미늘이 없는 기존 봉합사에 비해 우월하거나 동등한 결과를 보임 [PMID: 28603661].

복벽봉합시 피하드레인 삽입을 하는 것이 상처벌어짐을 감소시키는지에 대한 의견은 매우 다양함 [PMID: 19442311]. 몇몇 연구들은 피하드레인 삽입이 상처합병증을 감소시킨다고 보고함 [PMID: 24952366].

**2) 연구 가설 및 목적**

(1) Schema


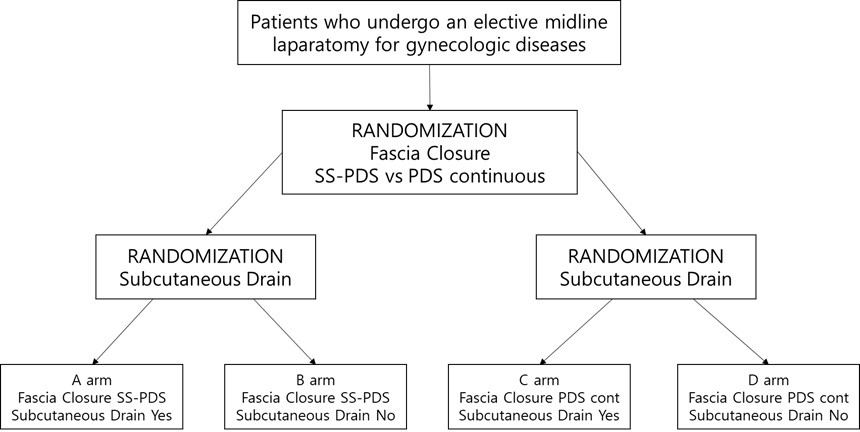


(2) 일차 목적

‘부인과질환으로 절중절개 개복술을 받는 여성에서 SS-PDS 를 사용하여 복부 근막봉합을 시행하는 것이 미늘이 없는 봉합사를 사용하는 것보다 수술 후 1년까지 절개창탈장 누적발생률을 감소시킬 것이다’ 는 가설을 증명하고자 함.

(3) 이차 목적

A. A+B군 vs C+D군에서 시간에 따른 절개창탈장의 발생곡선을 비교 (SS-PDS 여부에 따른 비교)

B. A+B군 vs C+D군에서 수술 후 4주까지 수술부위감염의 누적발생률과 종류를 비교 (SS-PDS 여부에 따른 비교)

C. A+B군 vs C+D군에서 수술 후 4주 상처벌어짐의 발생률, 누적발생률을 비교 (SS-PDS 여부에 따른 비교)

D. A+B군 vs C+D군에서 수술 후 2일째 측정된 간이통증조사지 (BPI-K) 점수를 비교 (SS-PDS 여부에 따른 비교)

E. A+B군 vs C+D군에서 수술 후 4일째 측정된 간이통증조사지 점수를 비교 (SS-PDS 여부에 따른 비교)

F. A+B군 vs C+D군에서 수술 후 4일까지 수집된 통증 NRS 점수를 비교 (SS-PDS 여부에 따른 비교)

G. A+C군 vs B+D군에서 수술 후 4주까지 수술부위감염의 누적발생률과 종류를 비교 (피하드레인 여부에 따른 비교)

H. A+C군 vs B+D군에서 수술 후 4주 상처벌어짐의 발생률, 누적발생률을 비교 (피하드레인 여부에 따른 비교)

I. A+B군 vs C+D군에서, A+C군 vs B+D군에서 절개창탈장, 통증, 수술부위감염, 상처벌어짐을 제외한 adverse event 의 발생률과 종류, grade, 연관성을 비교

J. SS-PDS 를 사용한 복부 근막봉합 수술비디오 제작

1. **임상시험용 의약품 및 의료기기 코드명(또는 주성분의 일반명), 원료약품의 분량, 제형 등(대조약 포함)**

**1) SS-PDS 실험군**

STRATAFIX Symmetric PDS Plus, suture size: 1/0, suture length: 45cm, needle size: 40mm (CT needle) (Ethicon, Somerville, NJ, USA)

**2) SS-PDS 대조군**

PDS Plus, suture size: 1/0, suture length: 90cm, needle size: 40mm (CT needle) (Ethicon, Somerville, NJ, USA)

**3) 피하드레인 실험군**

Jackson-Pratt drain, 7 Fr (제조사 미정)

**4) 피하드레인 대조군:** 해당 사항 없음

1. **연구대상자의 선정 기준, 제외기준, 목표한 대상자 수 및 산출 근거**
2. **선정기준**

(1) 부인과질환으로 정중절개 개복술을 받을 예정인 여성

(2) 나이 18세 초과

(3) ECOG performance status 0 - 2

1. **제외기준**

(1) 복부 절개창탈장이 이전에 있었거나 현재 있음

(2) 임신 중

(3) 골반 혹은 복부에 외부 방사선치료를 받은 경우

(4) PDS 성분에 알레르기가 있는 경우

(5) 조절되지 않는 당뇨, 자가면역 혈관염, 간경화 등 상처치유에 영향을 줄 수 있는 질환

(6) BMI>35

(7) 베바시주맙과 같이 상처치유에 영향을 줄 수 있는 약물을 사용하였거나 사용예정인 경우. 베바시주맙을 사용하는 경우 수술 전과 후 각 4주의 휴약기를 가지면 참여 가능함

(8) 최근 6개월 이내 복부 정중절개 수술을 받은 경우

(9) 감염이 의심되어 수술을 시행하는 경우

1. **목표한 대상자 수 및 산출 근거**

(1) 기존 연구 결과를 기반으로 SS-PDS 대조군 (C+D arm) 에서 1년 누적 절개창탈장 발생률은 0.13으로 추정 [PMID: 26188742].

(2) 복부 근막봉합에서 미늘봉합사를 사용한 후 절개창탈장 발생률을 조사한 기존 연구는 없음. 통상적인 봉합방법을 사용한 연구들에서도 1-2% 등 매우 낮은 절개창탈장 발생률을 보이는 경우가 있음을 고려하여 SS-PDS 실험군 (A+B arm) 에서 1년 누적 절개창탈장 발생률은 0.03 으로 추정함.

(3) Alpha 0.05 (one-sided), power 0.8, drop-out rate 0.06, each arm (A+B vs C+D): 87 patients

(4) 따라서, 목표한 대상자 수는 174 명임.

1. **연구 대상자 모집 계획**

연구자가 진료과정에서 접하는 환자들 중에서 연구대상자를 모집. 본 연구의 연구자들은 인종이나 사회경제적 상태에만 근거해서 이 연구에 참여할 가능성이 있는 환자를 배제시키지 않을 것임. 이 연구의 선정기준에 합당하다면, 가능한 환자들이 이 연구에 참여할 수 있도록 모든 노력을 다할 것이며 본 기관에서 부인과질환으로 정중절개 개복술을 받는 환자의 전체를 대표할 수 있도록 노력할 것임. 취약한 연구대상자는 연구 참여를 하지 않도록 할 것임

1. **연구 방법**
2. **Schema**

**
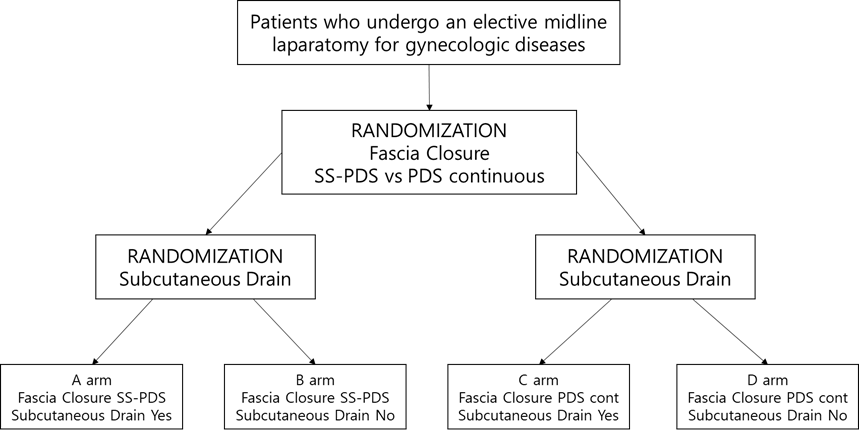
**

1. **비교군 설정 및 무작위 배정 방법**

(1) 서면 동의 취득

(2) 스크리닝번호 부여

A. 스크리닝을 시행하는 각 기관에서 부여. 각 기관에서 스크리닝번호가 중복되지 않도록 관리.

B. ‘S_XX_01’ 의 형태로 부여됨. XX 는 스크리닝을 시행한 기관의 이니셜임.

| 실시기관 명칭 (무순) | 기관 이니셜 |
| --- | --- |
| 분당서울대학교병원 | SB |
| 삼성서울병원 | SM |
| 국립암센터 | NC |
| 세브란스병원 | SV |
| 서울아산병원 | AM |
| 가천대 길병원 | GG |
| 아주대학교병원 | AJ |

(3) Eligibility check

A. 각 기관에서는 연구대상자가 연구 참여 가능한지 스크리닝 시행하고, eligibility check sheet 을 작성하여 서명하여 보관함.

B. 스크리닝 실패면 이후 단계 없이 스크리닝 실패로 처리함. 스크리닝 성공이면 eligibility check sheet 서명본의 스캔파일을 KGOG 로 송부

(4) Eligibility review 와 무작위배정

A. KGOG 에서는 각 기관에서 보낸 eligibility check sheet 서명본의 스캔파일을 확인한 후 불확실하거나 오류가 있으면 각 기관에 연락하여 명확히 함.

B. KGOG 에서 각 기관에서 보낸 eligibility check sheet 서명본의 스캔파일을 리뷰한 결과, eligible 하면 수술 전날 연구대상자가 입원할 때까지 무작위배정을 시행하지 않고 기다림. 무작위배정을 수술 전날에 시행하는 것은 수술 계획의 변동에 따른 중도탈락을 줄이기 위함임.

C. KGOG 에서 각 기관에서 보낸 eligibility check sheet 서명본의 스캔파일을 리뷰한 결과, ineligible 하면 각 기관에 통보하고 스크리닝 실패로 처리함.

D. 각 기관에서는 수술 전날 연구대상자가 수술을 위해 입원하였고, eligibility 에 변동이 없는 것이 확인되면 (수술방법 변경 등), KGOG 에 해당 사실을 알리고 무작위배정을 요청함. KGOG 에서 eCRF 시스템 (Redcap) 에 접속하여 eligibility sheet 을 전산 입력하고 무작위배정을 시행함. 랜덤번호는 ‘R_XX_01’ 의 형태로 부여됨. XX 는 기관의 이니셜임.

D. 무작위배정표는 미리 생성되어 eCRF 시스템에 내장되어 있음. 무작위배정표는 분당서울대학교병원 의학연구협력센터에서 미리 생성하여, 연구자를 거치지 않고 직접 eCRF 시스템에 import 될 것임. 무작위배정표의 구체사항은 아래와 같음

A) SS-PDS 실험군 vs 대조군의 비율은 1:1

B) 피하드레인 실험군 vs 대조군의 비율은 1:1

C) 층화 변수는 기관, BMI (≤30 vs >30), Surgery indication (Confirmed or suspected cancer vs not) 임. 층화 변수는 A), B) random 모두에 적용됨

D) Block randomization

(5) 무작위배정 결과의 통보

A. 무작위배정 결과는 수술 중 근막봉합이 시작되기 직전까지 통보되지 않아야 함

B. 수술 중 근막봉합이 시작되기 직전 각 기관에서 KGOG 에 전화로 스크리닝번호를 고지한 후 무작위배정 결과를 유선으로 확인함.

C. 무작위배정 결과는 0, 1 과 같은 숫자가 아닌 ‘SS-PDS 실험군’, ‘피하드레인 대조군’ 형식으로 통보되어야 함. 각 기관에선 무작위배정 결과를 전화로 통보 받은 직후, KGOG 측에 그 결과를 반복 말함으로써, 오류가 없도록 함.

D. KGOG 는 eCRF 에 랜덤번호에 따른 레코드를 생성하고, 스크리닝번호 – 랜덤번호 짝을 각 기관에 통보하여 각 기관에서 eCRF 에 자료를 입력할 수 있도록 함

(6) 무작위배정 결과의 은폐

A. SS-PDS 무작위배정 결과

A) 연구대상자에게 무작위배정 결과를 알리지 않음.

B) 연구자가 절개창탈장 진단시 무작위배정 결과에 영향을 받는 것을 최소화 하기 위해 무작위배정 결과는 의무기록에 기록하지 않고 CRF 에만 기록함.

B. 피하드레인 무작위배정 결과

무작위배정 결과를 은폐하지 않음

(7) 무작위배정 결과 은폐 해제

A. 해제 to 연구자: 별도의 승인 절차 없이 각 기관에서는 CRF 를 열람하여 무작위배정 결과를 확인할 수 있음.

B. 해제 to 연구대상자: 연구자가 의학적으로 필요하다고 판단하는 경우 연구자는 책임연구자의 승인을 거쳐 연구대상자에게 무작위배정 결과를 알려줄 수 있음.

(8) 중간 분석

A. 초기 안전성 검토를 위한 중간 분석

A) A+B 군에 10번째 연구대상자가 배정되어 4주째 방문을 마치면, A+B 군 10명의 연구대상자에 대해서 수술부위감염, 상처벌어짐, 그 외 adverse events 의 발생빈도와 종류, grade 를 평가할 예정임.

B) 평가 결과는 IRB 에 보고할 예정이며, 만약, adverse events 의 빈도, 종류, grade 가 통상적인 범위를 넘는다고 판단될 경우, 연구 중단을 포함하여 가능한 조치를 고려할 예정임.

B. 피하드레인의 상처벌어짐에 대한 중간 분석

A) A+C 군 vs B+D 군에서 각 군에 44번째 연구대상자가 4주째 방문을 종료하였을 때 시행

B) A+C 군의 상처벌어짐의 발생률이 B+D 군보다 낮으면 (one-sided p < 0.005), 피하드레인 삽입이 상처벌어짐을 감소시킨다고 판정하고, 피하드레인 삽입에 대한 무작위배정은 중단할 것임. 즉, 이후 모든 연구대상자에서 피하드레인을 삽입할 것임.

1. **시험약 투여∙사용량, 투여∙사용 방법, 병용 요법, 대조약 사용시 그 선택사유**

(1) SS-PDS 실험군 (SS-PDS 를 사용한 근막봉합)

A. 사용 방법

A) STRATAFIX Symmetric PDS Plus, suture size: 1/0, suture length: 45cm, needle size: 40mm (CT needle) (Ethicon, Somerville, NJ, USA)

B) 제조사에 의해 권고되고 기존 연구에 기술된 연속봉합 (running suture) 을 사용 [PMID: 28603661] (<https://www.youtube.com/watch?v=PTYtYvSaGMg>, <https://youtu.be/TPyulS4fQgQ>)

C) 복막 봉합을 해야 함. 복막제거술로 복막 봉합이 불가능한 경우는 하지 않을 수 있음

D) 근막봉합 시 5-8mm 간격으로, 절개면으로부터 양쪽으로 각각 5-8mm 두께의 조직을 포함하여 봉합을 시행함

E) 연구자의 판단에 따라, PDS 혹은 다른 성분의 봉합사를 사용하여 소수의 근막 비연속봉합 (interrupted sutures) 을 추가로 시행하는 것은 허용됨

F) 봉합사의 길이와 피부절개창 길이의 비율이 4 이상이 되도록 권고됨. 즉, 피부절개창의 길이가 20cm 이면 봉합사를 80cm 이상 사용하여 봉합하는 것이 권고됨

G) 직복근 (rectus muscle) 의 ventral 에 위치한 근막 (aponeurosis) 만 봉합하고, 과도한 지방조직이나 근육을 포함하여 봉합하지 않도록 함. Arcuate line 의 cephalic 쪽에서는 가능한 경우 직복근의 dorsal sheath 를 포함하여 봉합하는 것이 권고됨

H) 근막절개창의 cephalic 끝과 caudal 끝에서 각각 봉합을 시작하는 것이 권고됨. 즉, caudal 끝 (pubic bone에 닿은 근막 부분) 에서 봉합을 종료하지 말 것

I) 절개창의 가운데서 봉합이 종료할 경우, 권고된 대로 역방향으로 2-3번 봉합을 진행한 후 종료함. 또한, 다음 봉합사와 2cm 이상 봉합이 중복되도록 할 것 (부록 그림 1)

F) 필요한 경우 절개창의 한쪽 면에서 봉합을 시작할 수 있음 (부록 그림 1)

G) 일반적인 봉합사와 달리 미늘봉합사는 봉합 후 느슨해지지 않으므로, 과도하게 당겨 봉합을 하지 말것. 또한, 모든 stitch 에 고르게 압력이 배분되도록 일정한 힘으로 봉합을 시행할 것

B. 선택 사유

연구 배경에 기술하였음

(2) SS-PDS 대조군 (미늘봉합사가 아닌 통상적인 봉합사를 사용한 근막봉합)

A. 사용 방법

A) PDS Plus, suture size: 1/0, suture length: 90cm, needle size: 40mm (CT needle) (Ethicon, Somerville, NJ, USA)

B) 기존 연구에 기술된 연속봉합 (running suture) 을 사용 [PMID: 26188742]

C) 복막 봉합을 해야 함. 복막제거술로 복막 봉합이 불가능한 경우는 하지 않을 수 있음

D) 근막봉합 시 5-8mm 간격으로 절개면으로부터 양쪽으로 각각 5-8mm 두께의 조직을 포함하여 봉합을 시행함

E) 연구자의 판단에 따라, PDS 혹은 다른 성분의 봉합사를 사용하여 소수의 근막 비연속봉합 (interrupted sutures) 을 추가로 시행하는 것은 허용됨

F) 봉합사의 길이와 피부절개창 길이의 비율이 4 이상이 되도록 권고됨. 즉, 피부절개창의 길이가 20cm 이면 봉합사를 80cm 이상 사용하여 봉합하는 것이 권고됨

G) 직복근 (rectus muscle) 의 ventral 에 위치한 근막 (aponeurosis) 만 봉합하고, 과도한 지방조직이나 근육을 포함하여 봉합하지 않도록 함. Arcuate line 의 cephalic 쪽에서는 가능한 경우 직복근의 dorsal sheath 를 포함하여 봉합하는 것이 권고됨

H) 근막절개창의 양쪽 끝에서 봉합을 시작해도 되고, 한쪽 끝에서 시작해도 됨. 봉합사를 2개 사용할 경우 각각의 봉합사는 각각 매듭을 만들어 고정하며, 2cm 이상 봉합이 중복되도록 함

B. 선택 사유

A) 복부 정중절개 근막봉합의 방법은 비연속봉합 (interrupted sutures) 에서 모노필라멘트 봉합사를 이용한 연속봉합 (continuous running suture) 으로 발전해 옴. 최근 무작위배정 임상시험에서 조직을 작게 포함시키는 봉합법이 절개창탈장의 발생을 감소시킴 [PMID: 20395846,12594682, 26188742, 19917943].

B) 유럽탈장학회는 지연흡수 봉합사를 사용하여 복벽봉합을 권고하였고, 이것은 표준적 방법으로 받아들여지고 있음 [PMID: 25618025].

C) 2개의 메타분석에서 지연흡수 봉합사와 비흡수 봉합사를 비교하였을 때, 지연흡수 봉합사를 사용하였을 때 절개창탈장이 비슷하면서 suture sinus 와 상처통증이 감소하는 것에 기반하여 지연흡수 봉합사 사용을 권고함 [PMID: 20395846, 22061310].

D) 최근 메타분석에서 지연흡수 봉합사와 급속흡수 봉합사를 비교하였을 때 지연흡수 봉합사를 사용하는 경우 절개창탈장이 적었음 [PMID: 20395846]

(3) 피하드레인 실험군 (피하드레인의 삽입)

A. 사용 방법

A) 기기: Jackson-Pratt drain, 7 Fr (제조사 미정)

B) 밀폐 (closed), 음압 드레인을 피하에 거치. 드레인은 수술절개창과 별도의 피부 천공을 통해 삽입되어야 함

C) 피하조직 봉합은 불필요하나 연구자의 판단에 따라 시행할 수 있음

D) 피부봉합은 stapler, 비흡수성 봉합사를 사용한 봉합 중 각 기관의 통상적인 방법에 따라 시행

E) 피하드레인은 수술 후 3일 – 14일 사이에, 일일 배액량 10cc 미만에서 연구자의 판단에 따라 제거. 퇴원시까지 일일 배액량이 10cc 이상인 경우 연구자 판단에 따라 제거할 수 있음. 수술 후 14일이 지나도 일일 배액량이 10cc 이상인 경우 연구자 판단에 따라 제거하거나 유지할 수 있음

F) 피하층이 얇아 피하드레인 삽입이 불가능한 경우 연구자 판단에 따라 피하드레인을 삽입하지 않을 수 있음

B. 선택 사유

연구 배경에 기술하였음

(4) 피하드레인 대조군 (피하드레인을 삽입하지 않음)

A. 사용 방법

1. 피하드레인 삽입하지 않음

2. 피하조직 봉합은 불필요하나 연구자의 판단에 따라 시행할 수 있음

3. 피부봉합은 stapler, 비흡수성 봉합사를 사용한 봉합 중 각 기관의 통상적인 방법에 따라 시행

B. 선택 사유

연구 배경에 기술하였음

1. **관찰항목, 임상검사항목 및 관찰검사방법**

| 관찰항목 | 관찰검사방법 |
| --- | --- |
| 수술 후 1년까지 절개창탈장 누적발생률 | 연구대상자는 수술 후 1년째 외래 방문하도록 권고됨. 수술 후 9 - 15개월 사이 방문은 1년째 방문으로 간주할 수 있음. 연구자는 신체검진을 시행하여 절개창탈장을 진단함. 연구자는 수술 후 시행된 영상검사 결과를 참고할 수 있음. 절개창탈장 여부가 불확실한 경우, 확진을 위한 검사를 시행함. 연구대상자가 본 연구의 수술을 받은 후 다시 정중절개 수술을 받았거나, 사망하였거나, 추적 불가능한 경우 event 시점에서 censor 로 처리될 것임. 연구자는 군 배정에 대해 remind 되지 않을 것이며, 연구대상자는 1년째 방문이 종료될 때까지 군 배정에 대해 눈가림될 것임 |
| 수술 후 4주까지 수술부위감염의 누적발생률과 종류 | 연구대상자는 수술 후 4주째 외래 방문하도록 권고됨. 수술 후 3 – 5 주 사이 방문은 4주째 방문으로 간주할 수 있음. 연구자는 신체검진을 시행하여 수술부위감염을 진단함. 수술 후 4주째 방문 이전에 연구대상자가 외래를 방문하는 경우 연구자는 수술부위감염 여부와 종류에 대해 평가하여야 함. 수술 후 4주째까지 있었던 방문에서 얻어진 정보는 모두 수술부위감염 진단에 사용될 것임. 수술부위감염의 여부와 종류는 CDC 기준에 따라 판정될 것임 (https://www.cdc.gov/nhsn/PDFs/pscManual/17pscNosInfDef_current.pdf). CDC 기준에 따른 어떤 종류의 수술부위감염이라도 있다면 수술부위감염이 있다고 판정함. 연구자는 군 배정에 대해 remind 되지 않을 것임. |
| 수술 후 4주까지 상처벌어짐의 누적발생률 | 연구대상자는 수술 후 4주째 외래 방문하도록 권고됨. 수술 후 3 – 5 주 사이 방문은 4주째 방문으로 간주할 수 있음. 연구자는 신체검진을 시행하여 상처벌어짐을 진단함. 상처벌어짐은 모든 깊이, 모든 길이의 상처벌어짐으로 정의함. 수술 후 4주째 방문 이전에 연구대상자가 외래를 방문하는 경우 연구자는 상처벌어짐 여부에 대해 평가해야 함. 수술 후 4주째까지 있었던 방문에서 얻어진 정보는 모두 누적발생률 산정에 사용될 것임. 수술 후 4주째까지 상처벌어짐이 1번이라도 관찰된 경우, 4주째 스테이플러나 봉합의 제거가 불가능한 경우, 4주째 스테이플러나 봉합 제거한 후 추적에서 상처벌어짐이 발생한 경우는 모두 누적발생률에 산정됨 |
| 수술 후 4주 상처벌어짐의 발생률 | 연구대상자는 수술 후 4주째 외래 방문하도록 권고됨. 수술 후 3 – 5 주 사이 방문은 4주째 방문으로 간주할 수 있음. 연구자는 신체검진을 시행하여 상처벌어짐을 진단함. 수술 후 4주 상처벌어짐은 4주째 평가하였을 때 상처가 벌어짐, 스테이플러나 봉합의 제거가 불가능한 경우, 4주째 스테이플러나 봉합 제거한 후 추적에서 상처벌어짐이 발생한 경우로 정의함. 4주 이전에 상처벌어짐이 있었으나 4주째 평가하였을 때 벌어짐이 없는 경우는 수술 후 4주 상처벌어짐으로 판정하지 않음. 4주째 방문시 스테이플러나 봉합을 제거한 경우 1주 후 추적방문이나 전화상담이 필요함. 추적방문이나 전화상담에서 상처벌어짐이 없는 경우 수술 후 4주 상처벌어짐으로 판정하지 않음. 그러나, 추적방문이나 전화상담에서 상처벌어짐이 있는 경우 수술 후 4주 상처벌어짐으로 판정함 |
| 간이통증조사지 점수 | Baseline, 수술 후 2일, 4일째 BPI-Korean version 을 사용하여 측정 |
| 통증 NRS | 수술환자의 통상적인 모니터링인 NRS 로 측정하는 통증 점수와 기록 시간을 의무기록 리뷰를 통해 수술 직후로부터 수술 후 4일째 오후 10시까지 수집함 |
| 연구대상자, 수술 정보 | Date of birth, height, weight, current smoker, medical history, pre- and postoperative chemotherapy, corticosteroids, previous abdominal surgery, ASA classification, type of surgery, suture length, wound length, operation time, antibiotic prophylaxis, intra-abdominal drain, subcutaneous drain, DVT prophylaxis, Patient controlled analgesia (Y/N, type IV or epidural), wound anesthetics infiltration system, perioperative complications, transfusion, postoperative radiation, ICU care |
| 절개창탈장, 통증, 수술부위감염, 상처벌어짐을 제외한 adverse event 의 발생률과 종류, grade, 연관성 | 수술 입원 중, 수술 후 4주, 1년째 방문시 병력 청취와 신체검진을 통해 adverse event 를 수집함. 수술 후 1년째까지 발생한 모든 방문과 의료기록이 사용될 것임. Grade 는 CTCAE ver 4.0 을 사용함 |

1. **효과 평가기준, 평가 방법**

| 목적 | 분석, 평가 방법 | 분석 대상 코호트 |
| --- | --- | --- |
| A+B군 vs C+D군에서 수술 후 1년까지 절개창탈장 누적발생률 비교 | Cumulative incidence of incisional hernia of A+B arm will be compared with that of C+D arm using chi-square or Fisher's exact test. P value 0.05 will be used to determine significance. | All patients who undergo surgery. Patients who do not have incisional hernia will be censored at last visit. Incidence will be estimated from time-occurrence curve. |
| A+B군 vs C+D군에서 시간에 따른 절개창탈장의 발생곡선을 비교 | Time-occurrence curve of incisional hernia will be depicted using Kaplan-Meier method and be compared between A+B vs C+D arm using log-rank test. P value 0.05 will be used to determine significance. | All patients who undergo surgery |
| A+B군 vs C+D군에서 수술 후 4주까지 수술부위감염의 누적발생률과 종류를 비교 | Cumulative incidence of SSI of A+B arm will be compared with that of C+D arm using chi-square or Fisher's exact test. P value 0.05 will be used to determine significance. Type of SSI will be summarized. | Patients who undergo surgery |
| A+B군 vs C+D군에서 수술 후 4주 상처벌어짐의 누적발생률을 비교 | Cumulative incidence of wound dehiscence of A+B arm will be compared with that of C+D arm using chi-square or Fisher's exact test. P value 0.05 will be used to determine significance. | Patients who undergo surgery and complete week 4 visit |
| A+B군 vs C+D군에서 수술 후 4주 상처벌어짐의 발생률을 비교 | Incidence of wound dehiscence of A+B arm will be compared with that of C+D arm using chi-square or Fisher's exact test. P value 0.05 will be used to determine significance. | Patients who undergo surgery and complete week 4 visit |
| A+B군 vs C+D군에서 baseline, 수술 후 2, 4일째 측정된 간이통증조사지 (BPI-K) 점수를 비교 | BPI score (total and each question) obtained at baseline will be compared between A+B vs C+D arm. BPI score (total and each question) obtained at postoperative day 2 will be compared between A+B vs C+D arm. BPI score (total and each question) obtained at postoperative day 4 will be compared between A+B vs C+D arm. Student t-test will be used. P value 0.05 will be used to determine significance. If BPI score at baseline is imbalanced between arms, change of BPI score (from baseline to postoperative day 2, 4) will be compared. | Patient who undergo surgery and complete BPI |
| A+B군 vs C+D군에서 수술 후 4일까지 수집된 통증 NRS 점수를 비교 | NRS collected for postoperative 4 days will be compared between A+B vs C+D arm using linear mixed model. P value 0.05 will be used to determine significance. | Patients who undergo surgery and NRS are measured |
| A+C군 vs B+D군에서 수술 후 4주까지 수술부위감염의 누적발생률과 종류를 비교 (피하드레인 여부에 따른 비교) | Cumulative incidence of SSI of A+C arm will be compared with that of B+D arm using chi-square or Fisher's exact test. P value 0.05 will be used to determine significance. Type of SSI will be summarized. | Patients who undergo surgery |
| A+C군 vs B+D군에서 수술 후 4주 상처벌어짐의 누적발생률을 비교 (피하드레인 여부에 따른 비교) | Cumulative incidence of wound dehiscence of A+B arm will be compared with that of C+D arm using chi-square or Fisher's exact test. P value 0.05 will be used to determine significance. | Patients who undergo surgery and complete week 4 visit |
| A+C군 vs B+D군에서 수술 후 4주 상처벌어짐의 발생률을 비교 (피하드레인 여부에 따른 비교) | Incidence of wound dehiscence of A+C arm will be compared with that of B+D arm using chi-square or Fisher's exact test. P value 0.05 will be used to determine significance. | Patients who undergo surgery and complete week 4 visit |
| 절개창탈장, 통증, 수술부위감염, 상처벌어짐을 제외한 adverse event 의 발생률과 종류, grade, 연관성을 비교 | Incidence, type and grade of adverse events except incisional hernia, pain, SSI, wound dehiscence will be summarized using appropriate metrics. They will be compared using appropriate statisticial methods. | Patients who undergo surgery. |

1. **기존 치료 및 연구와의 차별점**

(1) 미늘봉합사를 사용하여 복부 근막봉합을 시행하는 것은 FDA, 대한민국 식품의약품안전처의 허가 사항 범위 내임

(2) 미늘봉합사를 사용하여 복부 근막봉합을 시행하는 것은 잠재적으로 절개창탈장을 감소시킬 가능성이 있음

(3) 미늘봉합사를 사용하여 복부 근막봉합을 시행한 체계적인 연구가 부족한 상태임

1. **연구대상자의 이익과 위험**

(1) 예측되는 부작용/위험 및 대처 방안

A. 예측되는 부작용/위험 – 인과성과 상관없이

본 연구는 정중절개 개복술을 받는 여성을 대상으로 함. 따라서, 수술에 수반되는 다양한 adverse events 가 관찰될 수 있음. 또한, 상당수의 연구대상자가 악성종양으로 진단, 치료 받을 것으로 예상되어, 그에 수반되는 adverse events 가 관찰될 수 있음

A) Blood and lymphatic system disorders: anemia, febrile neutropenia, hemolysis, leukocytosis

B) Cardiac disorders: palpitations, sinus bradycardia

C) Ear and labyrinth disorders: vertigo

D) Eye disorders: Blurred vision, conjunctivitis, dry eye

E) Gastrointestinal disorders: abdominal distension, abdominal pain, anal pain, ascites, bloating, constipation, diarrhea, dry mouth, dyspepsia, enterocolitis, fecal incontinence, flatulence, ileus, intra-abdominal hemorrhage, nausea, vomiting

F) General disorders and administration site conditions: chills, edema face, edema limb, fatigue, fever, infusion related reaction, infusion site extravasation, irritability, localized edema, non-cardiac chest pain, pain

G) Infections and infestations: abdominal infection, bladder infection, catheter related infection, peritoneal infection, pharyngitis, stoma site infection, urinary tract infection, wound infection

H) Injury, poisoning and procedural complications: intraoperative gastrointestinal injury, intraoperative venous injury, intraoperative urinary injury, large intestinal anastomotic leak, postoperative hemorrhage, wound complication, wound dehiscence

I) Investigations: activated partial thromboplastin time prolonged, alanine aminotransferase increased, alkaline phosphatase increased, aspartate aminotransferase increased, platelet count decreased, urine output decreased, weight gain, weight loss

J) Metabolism and nutrition disorders: anorexia, dehydration

K) Musculoskeletal and connective tissue disorders: back pain, flank pain, myalgia, neck pain

L) Psychiatric disorders: anxiety, delirium, insomnia

M) Renal and urinary disorders: hematuria, proteinuria, urinary frequency, urinary incontinence, urinary retention, urinary urgency

N) Reproductive system and breast disorders: menorrhagia, irregular menstruation, pelvic pain, vaginal discharge, vaginal dryness, vaginal hemorrhage

O) Respiratory, thoracic and mediastinal disorders: atelectasis, cough, dyspnea, hypoxia, pleural effusion, sore throat

P) Skin and subcutaneous tissue disorders: alopecia, pruritus, purpura, urticarial

Q) Vascular disorders: flushing, hot flashes, phlebitis, thromboembolic event

B. 미늘봉합사 사용에 따른 부작용/위험 및 대처 방안

A) 부작용/위험

미늘봉합사는 수년 전 부터 다양한 연부조직의 봉합에 사용되어 왔음. SS-PDS 는 복부 근막봉합에 사용하는 것이 허가되어 있음. 그러나, 아직까지 SS-PDS 를 사용하여 복부 근막봉합을 시행한 임상경험이 많지는 않은 상태임. 증가할 것으로 예측되지는 않으나, 발생 가능한 부작용/위험으로는 절개창탈장, 수술부위감염, 상처벌어짐, 통증의 증가가 있을 수 있겠음.

B) 대처방안

A+B 군에 10번째 연구대상자가 배정되어 4주째 방문을 마치면, A+B 군 10명의 연구대상자에 대해서 수술부위감염, 상처벌어짐, 그 외 adverse events 의 발생빈도와 종류, grade 를 평가할 예정임. 평가 결과는 IRB 에 보고할 예정이며, 만약, adverse events 의 빈도, 종류, grade 가 통상적인 범위를 넘는다고 판단될 경우, 연구 중단을 포함하여 가능한 조치를 고려할 예정임.

C. 피하드레인 사용에 따른 부작용/위험 및 대처 방안

피하드레인 사용은 표준적인 술식 중 하나로 간주될 수 있음. 따라서, 표준적 치료에 비해 추가되는 부작용/위험은 없다고 판단됨

(2) 예측되는 부작용/위험이 있는데도 연구를 수행해야 함에 대한 근거 (혹은 예상되는 과학적/의학적 이득) 기술

연구를 통해 예상되는 이득 (SS-PDS 를 사용하여 복부 근막봉합을 함으로써, 절개창탈장을 감소) 이 연구에 따른 부작용/위험 가능성 (SS-PDS 실험군에서 절개창탈장, 수술부위감염, 상처벌어짐, 통증의 증가) 보다 크다고 판단함

1. **중지∙탈락 기준**

**(1) 연구 중지**

A. 책임연구자는 SS-PDS 의 안전성이 낮다고 판단될 경우 연구 중지할 수 있음. 중간분석 결과가 나왔을 때, 심각한 adverse event 가 발생하였을 때 고려할 수 있음

B. 책임연구자는 IRB 의 권고에 의해 연구 중지할 수 있음

C. 책임연구자는 연구의 정상적 수행이 불가능한 경우 연구 중지할 수 있음

**(2) 연구대상자의 연구 참여 중단**

A. 연구대상자는 연구 참여 중에 조건 없이 연구 참여를 중단할 수 있음.

B. 정중절개 개복술을 받지 않은 경우 연구 참여 중단함. 이후 추적 관찰 없음.

C. 수술 후 4주 이후 절개창탈장이 진단된 경우, 이후 추적 관찰 없음. 이럴 경우 연구 참여 완료로 판정함. 수술 후 4주가 경과하지 않은 시점에서 절개창탈장이 진단된 경우, 수술 후 4주까지 추적관찰함.

D. 수술 후 복부 정중절개 개복술을 다시 받은 경우, 연구 참여 중단함. 이후 추적 관찰 없음

E. 연구대상자의 비협조로 더 이상 연구 참여가 불가능하다고 판단되는 경우, 책임연구자는 연구 참여 중단을 결정할 수 있음.

F. 연구 참여를 중단한 경우, 해당 시점까지 수집된 자료는 연구에 사용됨

1. **부작용을 포함한 안전성의 평가기준, 평가 방법 및 보고 방법**

**(1) 인과성의 평가**

A. 연구자가 SS-PDS 사용 (대조군 봉합사 포함) 과 인과성이 있음 vs 없음으로 판정. 인과성이 없다고 판정하기 어려운 경우는 모두 인과성이 있음으로 판정함

B. 피하드레인 사용에 관련하여서는 인과성을 평가하지 않음

**(2) 평가 방법**

A. A+C군 vs B+D군에서 SS-PDS 사용 (대조군 봉합사 포함) 과 인과성이 있는 adverse events 의 전체 빈도를 비교 (p-value 0.05 기준으로 유의성 판단)

B. A+C군 vs B+D군에서 SS-PDS 사용 (대조군 봉합사 포함) 과 인과성이 있는 adverse events 종류 별 빈도를 비교 (p-value 0.05 기준으로 유의성 판단)

C. A+C군 vs B+D군에서 SS-PDS 사용 (대조군 봉합사 포함) 과 인과성이 있는, grade 3 이상의 adverse events 의 전체 빈도를 비교 (p-value 0.05 기준으로 유의성 판단)

D. A+C군 vs B+D군에서 SS-PDS 사용 (대조군 봉합사 포함) 과 인과성이 있는, grade 3 이상의 adverse events 종류 별 빈도를 비교 (p-value 0.05 기준으로 유의성 판단)

**(3) 보고 방법**

A. 중대하고, 예상하지 못한, 인과성이 있는, SS-PDS 실험군에서 발생한 adverse event (SS-PDS SUSAR) : 연구자가 인지한 날로부터 7일 이내에 아래 조치를 시행한다.

A) 해당 기관 IRB 에 보고

B) Coordinating 책임연구자에게 보고: Coordinating 책임연구자의 위임을 받은 KGOG 는 보고를 받은 후 7일 이내에 식품의약품안전처에 보고하고 타 참여기관에 해당 내용을 전달하여 타 참여기관 IRB 에 해당 내용이 보고되도록 함

C) 단, 사망을 초래하거나 생명을 위협하는 경우에는 coordinating 책임연구자에게 즉시 보고

B. SS-PDS SUSAR 가 아닌 adverse event

A) Coordinating 책임연구자의 위임을 받은 KGOG 는 1년에 1회 모든 참여기관의 모든 adverse events 를 수집하여 모든 참여기관의 IRB 에 보고

B) SS-PDS SUSAR 가 아닌 adverse event 라도 연구자 판단하에 IRB 에 개별 보고할 수 있음. 이럴 경우 해당 내용을 coordinating 책임연구자에게 통보. Coordinating 책임연구자는 내용 검토 후 식약처 보고, 타 기관 IRB 보고 등 적절한 조치 시행

1. **자료안전성 모니터링 계획(DSMP)**

(1) 모니터링 계획

A. 모니터링 책임자: 대한부인종양연구회 CRA

B. On line 모니터링: 모니터링 책임자는 1달에 1회 eCRF 에 입력된 자료를 검토하고 불분명하거나 오류가 있는 내용에 대해 각 기관에 query 발송. 또한, 입력이 늦어지는 케이스에 대해 각 기관에 입력 요청

C. 대면 모니터링: 기관 별 4주째 방문을 완료한 연구대상자가 2명이 되었을 때와 10명이 되었을 때 방문 시행하여 KGOG 모니터링 SOP 에 따라 모니터링 시행

(2) 모니터링 빈도

On line 모니터링은 1달에 1회, 대면 모니터링은 연구기간 중 기관 별 2회

(3) 모니터링 결과 보고

A. KGOG 는 on line 모니터링을 시행한 후 (매달), 대면 모니터링을 시행한 후 모니터링 결과보고서를 작성하여 coordinating 책임연구자에게 보고함

B. Coordinating 책임연구자는 결과보고서를 해당 기관 책임연구자에게 송부하고, IRB 보고를 요청함.

C. 결과보고서에 위반 내용 등 별도로 IRB 보고가 필요한 사항이 있으면 coordinating 책임연구자는 해당 기관 책임연구자에게 해당 내용에 대한 IRB 보고를 요청함

D. Coordinating 책임연구자는 결과보고서 내용에 따라 개별 기관 책임연구자에게 Corrective plan 작성을 요청할 수 있음

1. **자료 분석 및 통계 분석 방법**

(1) 군간 단변수 비교분석에 관해서는 “효과 평가기준, 평가 방법” 과 “부작용을 포함한 안전성의 평가기준, 평가 방법 및 보고 방법” 에 기술하였음

(2) 연구대상자, 수술 정보 변수에서 군간 차이가 있을 경우 차이가 나는 변수를 포함하여 다변수 비교분석을 시행할 것임. 예를 들어 A+B 군 vs C+D 군 간에 나이 차이가 있는 경우 단변수 비교분석에 더해 나이를 포함한 다변수 분석을 추가로 시행할 것임.

1. **연구수행일정표**

Enrolment rate: 9/month

Submission date to health authority / ethics: March, 2020

Start of subject enrolment: August, 2020

End of Subject enrolment: March, 2022

End of Study: May, 2023

Report (as described in the contract, e.g. draft publication): Dec, 2023

Planned publication /presentation: Dec, 2023

1. **연구대상자의 안전보호를 위한 대책**
2. **연구의 윤리성 확보를 위한 기본 방안**

헬싱키 선언 (2013년 개정) 및 ICH-GCP를 준수할 것임

IRB 승인 후 연구를 수행하겠음

1. **연구대상자의 동의 과정**

(1) 연구대상자에게 설명하고 동의를 취득할 연구자: 김기동, 김용범, 노재홍, 서동훈, 김주현, 김주영, 황우연

(2) 동의를 제공할 자: 연구대상자

(3) 연구 설명 과정과 동의 취득 과정 사이의 대기 시간: 5분

(4) 강제 또는 부당한 영향의 가능성을 최소화시킬 방법: 연구에 참여하지 않더라도 어떠한 불이익도 없음을 연구대상자에게 명확히 설명하겠습니다

(5) 연구 설명 과정과 동의 취득 과정에서 연구자가 사용하는 언어: 한국어

(6) 연구대상자 또는 대리인이 이해할 수 있는 언어: 한국어

(7) 연구대상자 또는 대리인에게 제공되는 정보와 동의서 서식: 별개의 문서로 제출

1. **연구대상자의 보상 방안**

(1) 수술 후 4주, 수술 후 1년 방문 시 교통비로 방문 당 3만원을 지급함

(2) 연구 참여가 중단되면 중단 시점까지의 교통비를 지급함

(3) Baseline 방문, 수술 입원은 연구대상자의 수술과 관련된 방문으로 간주하여 교통비를 지급하지 않음

1. **연구대상자의 개인정보보호 방안**

(1) 환자의 의무기록번호 및 병리 번호는 책임연구자의 책임하에 별도의 파일로 보관하며 이를 코드화하여 연구데이터를 통하여 개인 신상 확인이 불가능하도록 관리

(2) 연구데이터는 패스워드로만 접근 가능한 eCRF 에 보관됨

(3) 생명윤리법 시행규칙 제15조에 따라 연구 관련 기록을 연구가 종료된 시점부터 3년간 보관하며, 보관기관이 지난 문서는 개인정보보호법 시행령 제16조에 따라 파기함

1. **취약한 연구대상자를 포함하는 경우 추가적인 보호조치 방안**

취약한 연구대상자는 연구에 참여하지 않음

1. **인체유래물의 보관 및 폐기 방법**

인체유래물 수집하지 않음

1. **참고 문헌**

연구계획서 본문 내에 Pubmed ID 로 삽입함

1. **SS-PDS 를 사용한 복부 근막봉합 수술비디오 제작**

1) 선택 동의 사항임

2) 사전에 동의를 한 연구대상자를 대상으로 SS-PDS 를 사용한 복부 근막봉합 술기를 비디오 제작함. 제작 시 개인정보나 민감한 신체부위가 촬영되지 않도록 해야 함

3) 사전에 동의를 한 피험자의 경우에도 비디오를 제작하지 않을 수 있음

4) 비디오가 제작된 경우 해당 사실을 연구대상자에게 수술 직후 고지함. 연구대상자가 비디오를 보기를 원하는 경우 연구자는 협조해야 함

**부록 그림 1**


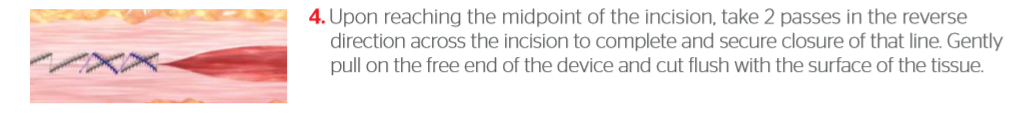


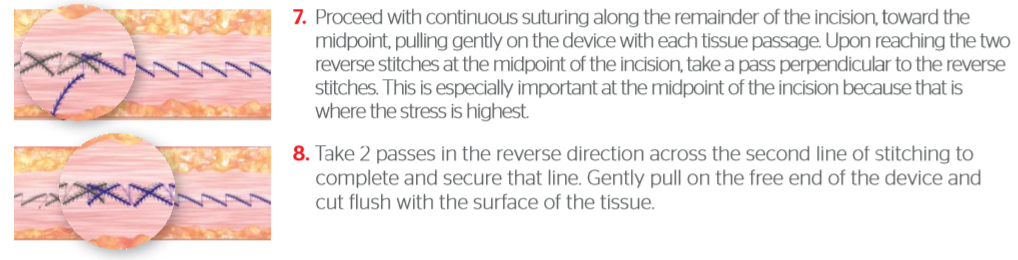


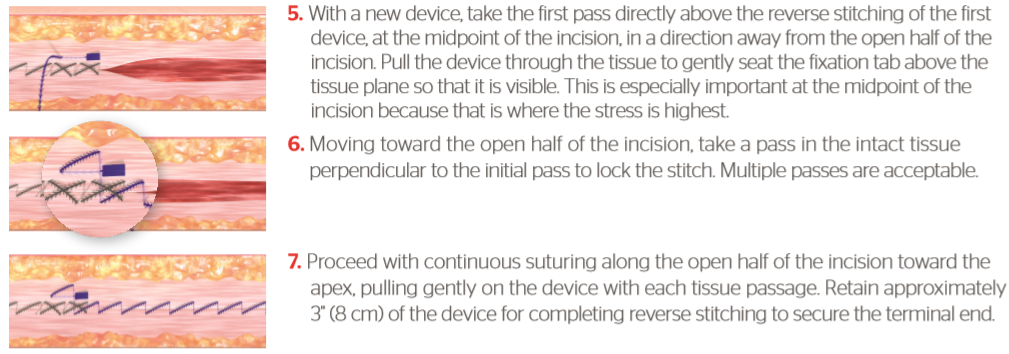

Supplement: S6 File — (DOCX) [file pone.0337036.s006.docx]
